# Biochar-based microbial fertilizer improves soil fertility and rice productivity by regulating soil nutrient-microbe-metabolite interactions

**DOI:** 10.3389/fmicb.2026.1802769

**Published:** 2026-03-30

**Authors:** Wendan Xiao, Dan Li, Qi Zhang, De Chen, Zhen Zhao, Miaojie Huang, Xiaolei Huang, Xuezhu Ye

**Affiliations:** 1 State Key Laboratory for Quality and Safety of Agro-Products, Key Laboratory of Soil Remediation and Quality Improvement of Zhejiang Province, Institute of Environmental Resources, Soil and Fertilizer, Zhejiang Academy of Agricultural Sciences, Hangzhou, China; 2 Hangzhou Agricultural Technology Extension Center (Hangzhou Plant Protection and Quarantine Center), Hangzhou, China

**Keywords:** bacterial community structure, biochar-based microbial fertilizer, functional microorganisms, rice productivity, soil fertility, soil metabolomics

## Abstract

Soil degradation and population growth threaten agricultural sustainability, necessitating innovative fertilization strategies. In this study, we developed and evaluated biochar-based microbial fertilizers (BMFs) that integrate biochar, organic manure, and functional microorganisms (*Bacillus subtilis, Bacillus megaterium, Azotobacter chroococcum*, and *Rhodopseudomonas palustris*) for their potential to enhance soil fertility and rice productivity. A greenhouse experiment demonstrated that BMFs application increased soil pH from 4.83 to 6.25–6.83, cation exchange capacity (CEC) by 19.61–38.26%, and organic matter (OM) by 24.92–43.69%. Significant increases were also observed in soil nutrient levels, with rises of 12.31–48.89% in ${\text{NH}}_{4}^{+}$-N, 30.02–69.81% in ${\text{NO}}_{3}^{-}$-N, 23.08–54.43% in available N, 29.72–85.24% in available P, and 22.45–50.40% in available K. These improvements in soil quality translated into promoted rice growth, as evidenced by increases in plant height, biomass, root length, and root surface area relative to the control. Beyond physicochemical properties, BMFs application also stimulated soil microbial biomass, enzyme activities, and diversity, while significantly altering microbial community structure. Redundancy analysis (RDA) confirmed that these structural shifts were primarily driven by the ameliorated soil conditions, including elevated pH, OM, CEC, and nutrient levels. High-throughput sequencing further indicated a notable enrichment of microbial genera taxonomically aligned with the inoculated functional strains. Integrated microbial and metabolomic analyses suggested synergistic mechanisms: the biochar–manure matrix contributed to soil improvement by neutralizing acidity, improving CEC, and increasing OM, while the introduced microbial consortium likely participated in nutrient cycling (N fixation, P/K solubilization, OM mineralization) and phytohormone production. Furthermore, microbial-metabolite network analysis revealed genus-specific associations (e.g., *Bacillus*-icosasphinganine, *Azotobacter*-eplerenone, *Rhodopseudomonas*-alatanin 2) that promoted growth-stimulating and stress-alleviating metabolites while suppressing root inhibitors and phytotoxins. Overall, BMFs offer a sustainable alternative to chemical fertilizers, mitigating soil degradation and enhancing rice productivity via synergistic biochar-microbe-metabolite interactions.

## 1 Introduction

Intensive agricultural practices, such as continuous cropping to maximize economic returns, have led to widespread soil degradation, compromising soil fertility and agricultural sustainability (Yang et al., 2024). Soil fertility, critical for agricultural productivity, supplies essential nutrients for crop growth but is diminished by degradation (Zhang et al., 2024). Chemical fertilizers have been widely used to supplement soil nutrients and enhance crop yields, playing a vital role in maintaining soil fertility in modern agriculture. However, their long-term and excessive application often leads to soil acidification and nutrient imbalances, necessitating sustainable alternatives (Fu et al., 2023; Yang and Zhang, 2023). Due to this, it is imperative that environmentally friendly, slow-release, and highly effective fertilizers be developed.

Biochar is a carbon-rich material that produced *via* biomass pyrolysis (300–800 °C) under low-oxygen conditions (Yan et al., 2021). As a soil amendment, biochar offers multiple agronomic and environmental benefits. It improves soil physical structure by reducing bulk density, enhancing porosity, and increasing water retention capacity (Gao et al., 2021). Chemically, biochar elevates soil pH in acidic soils, enhances cation exchange capacity (CEC), and effectively retains nutrients through its high surface area and adsorption capacity, thereby reducing nutrient leaching and preventing soil erosion (Wang et al., 2023; Xiao et al., 2022). Biologically, biochar provides a favorable habitat for soil microorganisms, modulates microbial community composition, and promotes beneficial microbial activities (Zhang et al., 2023). Additionally, biochar contributes to climate change mitigation by sequestering stable carbon in soil and reducing greenhouse gas emissions (Xiao et al., 2024). Despite these advantages, biochar alone contains limited available nutrients and cannot fully satisfy crop nutrient demands, which limits its performance as a standalone fertilizer (Gao et al., 2021). To overcome this limitation, biochar is increasingly integrated with organic or mineral fertilizers to develop biochar-based fertilizers, which have been shown to synergistically improve soil structure, nutrient use efficiency, and carbon sequestration capacity (Ren et al., 2023; Yang et al., 2024).

Soil microbiota drive key nutrient cycling processes, including nitrogen (N) fixation and phosphorus (P)/potassium (K) solubilization (Liu et al., 2024; Wei et al., 2024). Microbial fertilizers can enhance plant uptake of N, P, and K, thereby facilitating plant growth (Li et al., 2023). Among various functional microbes, the genus *Bacillus* is widely used in microbial fertilizers due to its strong stress resistance, plant growth-promoting effects, and disease-suppressive capacity (Wang et al., 2023). Specifically, *Bacillus subtilis* contributes to organic matter (OM) decomposition, biological N fixation, and plant growth stimulation (Fu et al., 2023), while *Bacillus megaterium* secretes organic acids and phosphatases that solubilize insoluble phosphates, increasing P bioavailability (Qi et al., 2023; Zhao et al., 2021). *Azotobacter chroococcum* is a free-living N-fixing bacterium that can enhance plant-available N in the rhizosphere (Li et al., 2024). Photosynthetic bacteria, such as *Rhodopseudomonas palustris*, can enhance plant photosynthetic efficiency and biomass production (Wang D. W. et al., 2022), and this species has been successfully used in microbial fertilizers to boost crop growth (Lee et al., 2016; Xu et al., 2018). Given these complementary functional traits, we hypothesized that integrating these four species into a biochar-based fertilizer could synergistically improve soil fertility and crop productivity. Accordingly, we investigated the ability of *Bacillus subtilis, Bacillus megaterium, Azotobacter chroococcum*, and *Rhodopseudomonas palustris* to enhance soil fertility and promote rice growth when formulated as a biochar-based microbial fertilizer (BMF).

Under field conditions, pure microbial fertilizers are becoming practically inefficient due to the low surviving ability of microbes in soil for an extended period of time, necessitating the use of potent carriers extending their thriving capacity in crop fields (Monica et al., 2025; Vassilev et al., 2020). During recent years, biochar due to its high surface area and porosity has been emerging as an ideal carrier for these microbes, forming biochar-based microbial fertilizers (BMFs) that enhance microbial survival and activity (Ren et al., 2023; Wang et al., 2023). BMFs employ constitutive potential of biochar and microbial activity to improve soil health and fertility, offering a sustainable alternative to chemical fertilizers for improving crop yields.

Rice, a staple for ~40% of the global population, faces yield declines due to soil degradation (Zhang et al., 2024). To boost rice yields, extensive application of chemical and organic fertilizers is common; however, chemical fertilizers contribute to soil acidification (Yang and Zhang, 2023), while organic fertilizers exacerbate greenhouse gas emissions (Xiao et al., 2024). Consequently, BMFs are urgently needed as a promising alternative strategy to address these issues (Bello et al., 2023; Ren et al., 2023; Yan et al., 2021). Biochar has gained attention as a soil amendment due to its ability to improve soil structure, enhance nutrient retention, and provide a habitat for microorganisms. However, biochar alone contains limited available nutrients and may not sufficiently support the survival and activity of functional microorganisms. Therefore, the incorporation of organic manure as a nutrient-rich carrier is essential to provide an energy source for microbial proliferation and to enhance the overall efficacy of biochar-based microbial fertilizers (Bello et al., 2023; Wang D. et al., 2022). While biochar-based fertilizers show promise, their integration with both functional microbes and organic manure—forming a biochar-based microbial fertilizer (BMF)—remains underexplored, particularly regarding mechanisms enhancing soil fertility and rice productivity. Unlike biochar-based organic fertilizers that rely solely on nutrient supply from organic sources, or biochar-based microbial fertilizers that may lack an organic carrier, our BMF integrates all three components (biochar, organic manure, and functional microbes) to synergistically improve soil health. We hypothesized that this combined system would restructure soil microbial communities and metabolite profiles, thereby alleviating soil degradation and enhancing rice growth.

Hence, the objectives of this study were to: (i) develop a novel and highly effective BMF that can replace conventional fertilizers by integrating biochar, functional microbes, and organic manure; (ii) evaluate its effects on key soil fertility parameters and rice growth performance; and (iii) elucidate the complex microbial and metabolic mechanisms underlying the regulation of soil fertility and rice growth. Our findings will advance the development of sustainable fertilizers and clarify their mechanisms for enhancing soil health and promoting sustainable rice production.

## 2 Materials and methods

### 2.1 Materials

The soil used in this study was air-dried and passed through a 2 mm sieve prior to use. All soil physicochemical properties were presented on an oven-dry weight basis. The basic physicochemical properties of the soil used in this study are as follows: pH 4.83, total organic matter (OM) 15.62 g kg^−1^, total N 0.62 g kg^−1^, total P 0.56 g kg^−1^, total K 15.62 g kg^−1^, available N 23.65 mg kg^−1^, available P 6.89 mg kg^−1^, and available K 76.26 mg kg^−1^.

Corn straw biochar was produced *via* pyrolysis (450 °C, 5 h) in a tube furnace under a continuous nitrogen gas (N_2_) flow (200 mL min^−1^), following established methods (Xiao et al., 2024; Yang C. et al., 2021). The biochar had a pH of 9.63, surface area of 45.6 m^2^ g^−1^, ash content of 28.6%, and CEC of 26.2 cmol kg^−1^.

The organic manure comprised sheep manure, chicken manure, and rice husk (2:1:1 ratio). The physicochemical properties were: pH 8.52, OM 656.3 g kg^−1^, total N 18.1 g kg^−1^, total P 13.4 g kg^−1^, total K 17.1 g kg^−1^.

*Bacillus subtilis, Bacillus megaterium, Azotobacter chroococcum*, and *Rhodopseudomonas palustris* were isolated and purified in our laboratory. The four bacterial strains were originally isolated from agricultural soils using standard serial dilution and spread-plate techniques on selective media. For *Bacillus* species, nutrient agar was used, and isolates were identified based on colony morphology, Gram staining, and biochemical tests, followed by 16S rRNA gene sequencing and BLAST analysis (Aehsas et al., 2025). *Azotobacter chroococcum* was isolated using Winogradsky medium, and purified and identified based on Gram staining, pigmentation, cell morphology, and cyst formation, followed by confirmation via 16S rRNA gene sequencing (Khosravi and Dolatabad, 2020). *Rhodopseudomonas palustris* was isolated using Biebl and Pfennig's medium under anaerobic conditions, and identified based on cultural, morphological and biochemical characteristics, followed by 16S rRNA gene sequencing for molecular confirmation (Nalvothula et al., 2022). These bacteria were cultured in Luria-Bertani broth medium at 28 °C to a concentration of 10^8^ CFU mL^−1^ for pot experiments.

### 2.2 Preparation and characterization of BMFs

BMFs were prepared by mixing biochar with bacterial suspensions and organic manure. The optimal component ratios were established through two-step preliminary experiments. First, to ensure sufficient nutrient supply for bacterial survival and proliferation, different volumes of bacterial fermentation suspension (1 × 108 CFU mL^−1^) were mixed with organic manure. The 1:100 ratio was found to be optimal, providing adequate nutrients for microbial growth. Second, this inoculated organic manure was then combined with biochar at various ratios to evaluate the coating efficiency and cost-effectiveness. The 3:1 ratio (inoculated manure: biochar) was selected as it achieved effective coating and uniform distribution while minimizing biochar input and production costs. Specifically, 12 kg of organic manure were initially mixed with 120 ml of bacterial fermentation suspension (1 × 10^8^ CFU mL^−1^) to supply nutrients. Then this mixture was combined with 4 kg of biochar in a coating machine and mixed for 30 min until fully incorporated. Five BMF types were prepared, containing *Bacillus subtilis* (BS), *Bacillus megaterium* (BM), *Azotobacter chroococcum* (AC), *Rhodopseudomonas palustris* (RP), or a combination of all four (CM). The plate dilution method assessed bacterial loading in BMFs. After 24 h of culturing at 35 °C on agar, bacterial counts ranged from 4.3 × 107 to 8.5 × 107 CFU g^−1^ (Supplementary Figure S1). The basic physicochemical properties of the five BMFs were measured, and the results are summarized in Table 1.

**Table 1:** Physicochemical properties of the five biochar-based microbial fertilizer (BMF) types.

BMF type	pH	Organic matter (g kg^−1^)	Total N (g kg^−1^)	Total P (g kg^−1^)	Total K (g kg^−1^)
BS	8.78 ± 0.12	492.6 ± 18.6	14.62 ± 0.82	12.56 ± 1.02	15.23 ± 1.57
BM	8.69 ± 0.15	486.5 ± 15.3	14.37 ± 1.15	11.19 ± 0.98	14.97 ± 0.85
AC	8.74 ± 0.09	489.4 ± 22.6	13.28 ± 1.26	11.35 ± 0.75	15.94 ± 0.88
RP	8.81 ± 0.18	475.6 ± 19.7	15.16 ± 0.93	12.18 ± 1.93	16.25 ± 1.26
CM	8.79 ± 0.11	498.5 ± 24.7	14.98 ± 1.05	12.69 ± 1.28	16.93 ± 1.78

### 2.3 Pot experiment design

A pot experiment was conducted to evaluate the effectiveness of BMFs in improving soil fertility and promoting rice growth. Six treatments were tested in triplicate: (1) CK: unamended control; (2) BS: *Bacillus subtilis* loaded BMF; (3) BM: *Bacillus megaterium* loaded BMF; (4) AC: *Azotobacter chroococcum* loaded BMF; (5) RP: *Rhodopseudomonas palustris* loaded BMF; (6) CM: BMF loaded with combined bacteria.

Each pot was filled with 5 kg of soil. For the BMF treatments (BS, BM, AC, RP, and CM), the corresponding BMF was applied at a rate of 10 g kg^−1^ soil (50 g per pot) and thoroughly mixed with the soil prior to rice transplanting. The CK treatment received no BMF amendment. In addition, all pots received basal inorganic fertilizers at rates of 150 mg N kg^−1^, 30 mg P kg^−1^, and 75.5 mg K kg^−1^, which were thoroughly mixed with the soil before transplanting.

The rice cultivar used in this study was *Oryza sativa* L. cv. “Yongyou 15”, a commonly grown hybrid variety in the local region. Four germinated seedlings were transplanted into each pot, and all pots were randomly arranged in a greenhouse with three replicates per treatment. Throughout the cultivation period, the rice plants were maintained under an alternating wet-dry irrigation regime: the pots were initially flooded with approximately 2 cm of water above the soil surface, then allowed to dry naturally for 2–3 days until small cracks appeared, at which point they were reflooded. This cycle was repeated throughout the experiment.

### 2.4 Sampling and analysis

#### 2.4.1 Sampling and analysis of physicochemical properties

Measurements of plant height, root length, and fresh weight were conducted after 50 days of rice growth. Plants were uprooted and separated into aboveground parts and roots, oven-dried, and weighed for dry weight, followed by post-harvest soil sample collection. Three soil cores were collected from each pot using a steel auger (2.0 cm diameter × 15 cm depth) and homogenized to form one composite sample. Each soil sample was divided into three sub-samples. One fresh sub-sample was used to determine microbial biomass C/N, ammonium nitrogen (${\text{NH}}_{4}^{+}$-N), nitrate nitrogen (${\text{NO}}_{3}^{-}$-N), available phosphate, available potassium, and five enzyme activities (dehydrogenase, urease, sucrase, amylase, and alkaline phosphatase) in 3 days. The second sub-sample was stored at −80 °C for microbial DNA analysis. As a third subsample, the remaining sample was further freeze-dried and subsequently screened through 10- or 100-mesh sieves for physicochemical analyses. Measurement details are provided in Supplementary material Section 1 for all soil physicochemical properties, including pH, OM, CEC, ${\text{NH}}_{4}^{+}$-N, ${\text{NO}}_{3}^{-}$-N, and available N, P, and K.

#### 2.4.2 Soil microbial biomass and enzyme activity

Microbial biomass C and N were measured using the fumigation–extraction method (Wang D. et al., 2022). Following chloroform fumigation, dissolved C and N in the potassium sulfate (K_2_SO_4_) extracts were quantified using a TOC Analyzer (TOC-VCSH, Shimadzu, Japan). Activities of the five soil enzymes (dehydrogenase, urease, sucrase, amylase, and alkaline phosphatase) were measured using enzyme kits (Sino Best Biological Technology Co., Ltd., Shanghai, China) following given instructions.

#### 2.4.3 16S rRNA gene amplification and sequencing

Soil genomic DNA was extracted using the Fast Soil DNA Kit (Omega Biotek, Inc.). DNA concentration and purity were assessed using agarose gel and a Nanodrop spectrophotometer (Thermo Scientific, DA, USA). The V3-V4 region of the *16S rRNA* gene was used as the bacterial-specific fragment with the following primers: 341F 5′-CCTACGGGNGGCWGCAG3′) and 806 R 5′-GGACTACHVGGGTATCTAAT 3′). High-throughput sequencing technique was used to analyzed the amplified *16 S rRNA* genes. Briefly, paired-end sequencing was performed on an illumina platform (GeneDenovo Biotechnology Co., Ltd., Guangzhou, China). All samples were rarefied to an equal sequencing depth of 80,000 reads per sample prior to diversity analyses. Rarefaction curves confirmed that sequencing depth was sufficient to capture the majority of microbial diversity. Raw sequencing data were processed using the DADA2 method in QIIME 2 to obtain amplicon sequence variants (ASVs). Details of amplification, sequencing, and bioinformatics analysis are provided in Supplementary material Section 2.

#### 2.4.4 Soil metabolite analysis

Non-targeted metabolomics was used to quantify the effects of BMF on soil metabolites. Soil samples were extracted using an acetonitrile and methanol mixture (v/v = 1:1). Metabolites were quantified using ultra-high performance liquid chromatography Q-Exactive mass spectrometry (details in Supplementary material Section 3). Significant metabolite differences were identified using partial least squares discriminant analysis (PLS-DA) with VIP > 1 and *p* < 0.05. Differential metabolites were analyzed for metabolic enrichment and mapped to biochemical pathways.

### 2.5 Statistical analyses

Results are presented as mean ± standard deviation of three replicates. All data were tested for normality using the Shapiro-Wilk test. For normally distributed variables, one-way ANOVA with the least significant difference (LSD) post-hoc test was performed. For non-normally distributed variables, the Kruskal–Wallis rank sum test followed by Dunn's test was used. Raw sequencing data were analyzed with the DADA2 method in QIIME 2 to generate ASVs. Taxonomic annotation was performed based on the SILVA database. After rarefying samples to a uniform sequencing depth, alpha diversity indices were calculated. PCoA was used to visualize beta diversity based on Bray-Curtis dissimilarity, and LEfSe was applied to analyze differentially abundant taxa among all groups.

## 3 Results

### 3.1 Effects of BMFs on soil properties

**Figure 1:**
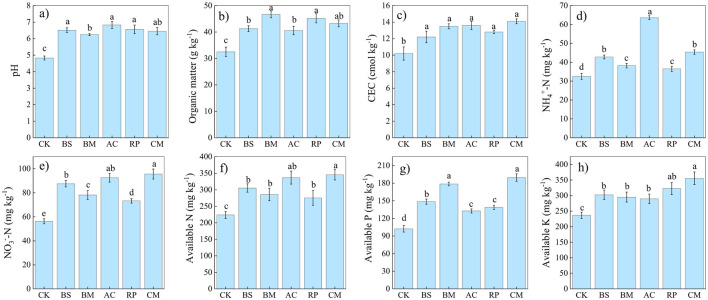
Effects of biochar-based microbial fertilizers on soil properties, including pH **(a)**, organic matter **(b)**, CEC **(c)**, ${\text{NH}}_{4}^{+}$-N **(d)**, ${\text{NO}}_{3}^{-}$-N **(e)**, available N **(f)**, available P **(g)**, and available K **(h)**. Different lowercase letters above the columns indicate significant differences among treatments at the *p* < 0.05 level.

The effects of different types of BMFs on soil physicochemical properties are illustrated in Figure 1. With respect to soil acidity, BMFs significantly increased soil pH from 4.83 to 6.25–6.83. BMFs-amended treatments also enhanced soil CEC by 19.61–38.26% as compared with CK. Soil OM increased by 8.2 to 14.2 g kg^−1^ as compared to CK, reaching a maximum of 46.7 g kg^−1^ at the BM treatment, when biochar was combined with *Bacillus megaterium*.

BMFs also significantly improved soil nutrient profile. The concentrations of ${\text{NH}}_{4}^{+}$-N, ${\text{NO}}_{3}^{-}$-N, available N, available P, and available K were all significantly enhanced under BMFs treatments (BS, BM, AC, RP, and CM, *p* < 0.01). Specifically, BS, BM, AC, RP, and CM treatments significantly increased the amount of ${\text{NH}4}_{4}^{+}$-N as compared to CK, with AC showing the greatest increase of 95.69%. Additionally, ${\text{NO}}_{3}^{-}$-N content in BS, BM, AC, RP, and CM treatments exhibited significant increases compared to CK, with increases of 55.42%, 38.89%, 64.30%, 30.02%, and 69.81%, respectively. Available N content increased by 36.54%, 27.73%, 50.58%, 23.08%, and 54.43% following the amendment of the BS, BM, AC, RP, and CM. Similarly, available P contents increased by 45.26%, 74.58%, 29.72%, 35.58%, and 85.24%, respectively, through amendment of BS, BM, AC, RP, and CM. Furthermore, notable increases were observed in available K contents (BS 27.95%, BM 24.86%, AC 22.45%, RP 36.36%, CM 50.40%).

### 3.2 Effects of BMFs on rice growth

**Figure 2:**
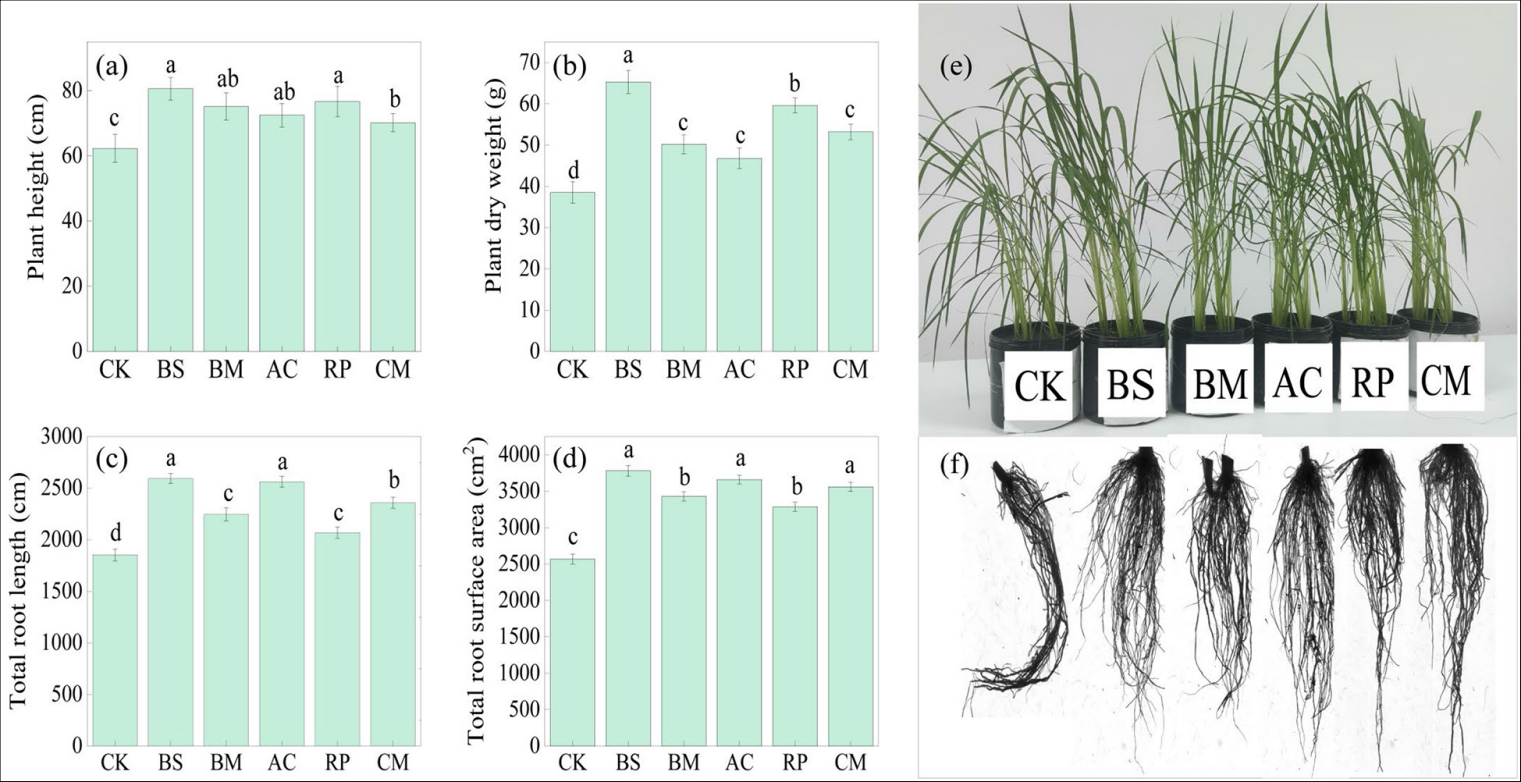
Effects of biochar-based microbial fertilizers on rice growth. **(a)** plant height; **(b)** plant dry weight; **(c)** total root length; **(d)** total root surface area; **(e)** Plant growth image at the fourth weekend; **(f)** plant root image. Different lowercase letters above the columns indicate significant differences among treatments at the *p* < 0.05 level.

BMFs-amended treatments significantly increased plant height and dry weight by 12.68–29.37% and 21.56–69.61%, respectively, compared with the CK group (Figures 2a, b). Root traits were also markedly improved, with total root length and total root surface area increasing by 11.71–40.01% and 27.94–47.15%, respectively (Figures 2c, d). Figures 2e,f presented the promoting effects of BMFs on rice aboveground and root growth. Such enhancement in root architecture, characterized by increased length and surface area, is indicative of a more extensive root system for nutrient and water acquisition. This indicates that BMFs enhance rice growth not only by directly providing nutrients, but also by fundamentally boosting the plant's ability to utilize soil resources.

### 3.3 Effect of BMFs on soil microbial biomass and enzyme activity

Soil microbial biomass is a key indicator of soil quality (Wang D. et al., 2022). The application of BS, BM, AC, RP, and CM significantly increased soil microbial biomass C by 128.12 %, 86.48%, 90.22%, 53.72%, and 123.34 %, respectively (Table 2). Similarly, microbial biomass N increased by 84.90%, 65.32%, 73.98%, 39.71%, and 73.94%, respectively.

**Table 2:** Soil microbial biomass (C and N), and enzyme activity (dehydrogenase, urease, sucrase, amylase, and acid phosphatase) under different treatments.

Treatments	Soil microbial biomass		Soil enzyme activity				
	C mg C kg^−1^ soil	N mg N kg^−1^ soil	Dehydrogenase μg g^−1^ soil d^−1^	Urease μg g^−1^ soil d^−1^	Sucrase mg g^−1^ soil d^−1^	Amylase mg g^−1^ soil d^−1^	Alkaline phosphatase μmol g^−1^ soil d^−1^
CK	433.57 ± 15.02 d	52.45 ± 3.26 d	58.19 ± 2.35 d	52.09 ± 3.36 d	5.63 ± 0.25 c	5.13 ± 0.31 c	1.75 ± 0.15 b
BS	989.04 ± 25.6 a	96.98 ± 2.98 a	108.91 ± 5.39 a	238.17 ± 8.28 a	9.13 ± 0.39 b	9.31 ± 0.45 b	3.12 ± 0.23 a
BM	808.52 ± 22.3 b	86.71 ± 2.39 b	94.21 ± 3.58 b	175.41 ± 6.24 c	11.99 ± 0.35 a	10.21 ± 0.52 b	3.74 ± 0.35 a
AC	824.72 ± 15.8 b	91.25 ± 2.89 b	93.26 ± 4.52 b	199.03 ± 6.96 b	11.87 ± 0.52 a	11.83 ± 0.56 ab	3.55 ± 0.28 a
RP	666.48 ± 36.1 c	73.28 ± 3.25 c	82.53 ± 5.26 c	186.57 ± 5.25 bc	6.28 ± 0.45 c	13.25 ± 0.73 a	3.25 ± 0.18 a
CM	968.33 ± 28.9 a	91.23 ± 2.96 ab	120.28 ± 6.38 a	254.13 ± 8.76 a	11.22 ± 0.56 a	13.06 ± 0.66 a	3.63 ± 0.24 a
*F*-values							
Treatments	89.32^**^	68.76^**^	123.57^**^	156.88^**^	55.63^**^	68.23^**^	32.56^**^

Values are presented as mean ± standard deviation (SD) (*n* = 3). Asterisks indicate a significant overall effect of treatment: ^*^p < 0.05, ^**^p < 0.01.

Soil enzymes, derived mainly from plant root exudates, microbial secretions, and decomposition residues, play essential roles in OM decomposition, nutrient mineralization, and the catalysis of soil biochemical reactions (Wang et al., 2023). BMFs application significantly enhanced the activity of five key enzymes (dehydrogenase, urease, sucrase, amylase, and alkaline phosphatase) as shown in Table 2. Compared with the CK, all BMFs treatments significantly enhanced soil dehydrogenase, urease, sucrase, amylase, and alkaline phosphatase activities. Specifically, all BMFs effectively promoted dehydrogenase activity. CM exerted the strongest stimulatory effect on urease activity, followed by the other BMF treatments. BM and AC showed the most pronounced enhancement of sucrase activity, while the other BMFs also promoted this enzyme to a notable degree. RP and CM exhibited the most marked positive effects on amylase activity. All BMFs treatments clearly increased alkaline phosphatase activity. These results indicate that BMFs application can effectively improve soil enzymatic activities related to C, N, and P cycling, thereby enhancing soil microbial metabolic capacity.

### 3.4 BMFs reshape the rice rhizosphere soil microbial community

#### 3.4.1 Microbial diversity

**Figure 3:**
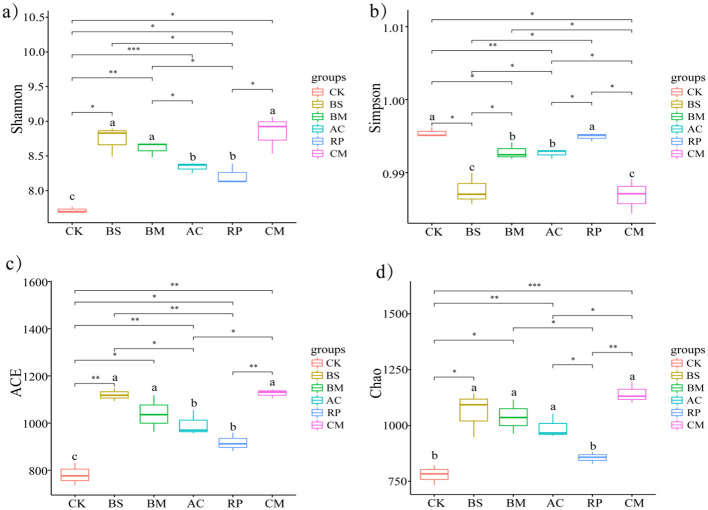
Effects of biochar-based microbial fertilizers on microbial diversity indices. **(a)** Shannon, **(b)** Simpson, **(c)** Ace, and **(d)** Chao indices. The significance of differences was determined using the paired *t*-test. *p < 0.05, **p < 0.01, ***p < 0.001, and ns, no significance. Different lowercase letters indicate significant differences among treatments (*p* < 0.05).

Soil microbial communities, as vital components of soil ecosystems, are closely linked to soil biogeochemical processes and serve as indicators of soil fertility status (Ren et al., 2023; Yang and Zhang, 2023). Alpha-diversity within microbial communities was quantified using the Shannon and Simpson indices for diversity, and the ACE and Chao1 indices for richness (Figure 3). A high Shannon index value and a low Simpson index value correspond to greater diversity (Xiao et al., 2024). Consequently, the Simpson index decreased, while the Shannon index increased in BMFs amended soils, indicating an increase in bacterial community diversity. The combined bacteria (CM) treatment demonstrated the most significant improvement. BS, BM, and AC all showed significant improvements in microbial diversity, whereas RP exhibited weakest effects. Additionally, ACE and Chao values demonstrated consistent variation trends, with both indices being enhanced by BMFs amendments. CM and BS showed the greatest improvement effect, followed by BM, AC, and RP.

#### 3.4.2 Microbial community structure

**Figure 4:**
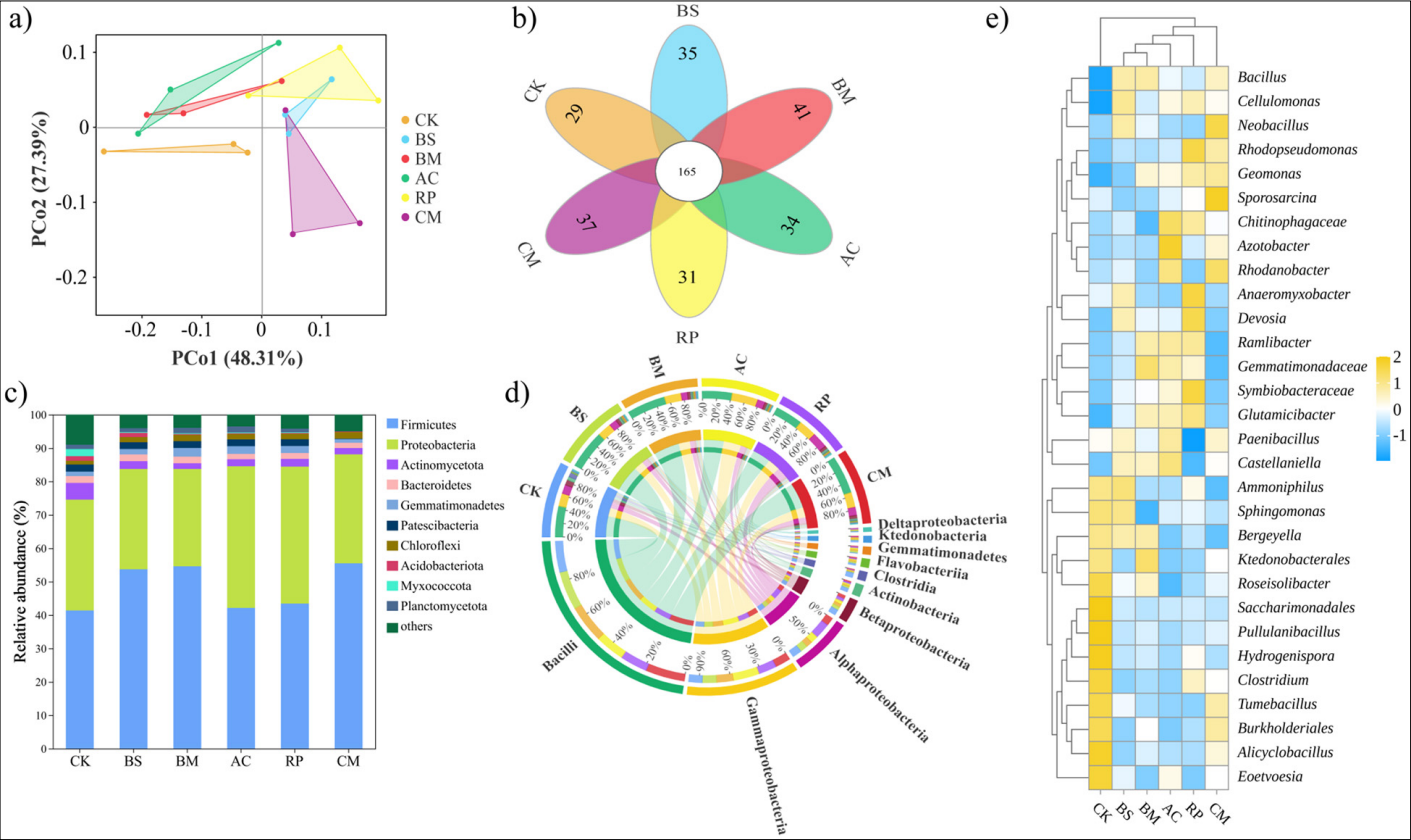
The response of microbial communities to biochar-based microbial fertilizer amendments. **(a)** Principal coordinate analysis (PCoA); **(b)** Venn diagram showing shared and unique ASVs; **(c)** Relative abundances (%) of the major bacterial phyla; **(d)** Circos diagram illustrating correlations between treatments and abundances of different bacterial classes; **(e)** Hierarchically clustered heatmap showing the relative abundance of dominant bacterial genera across all treatments. The color scale represents Z-score-normalized relative abundance, where positive values (yellow) indicate enrichment and negative values (blue) indicate depletion of a genus relative to its mean abundance across all samples.

BMFs application induced noticeable shifts in soil microbial community structure, as demonstrated by multivariate analysis (Figure 4). Principal coordinate analysis (PCoA) revealed distinct clustering patterns among treatments, with the first two principal coordinates (PCo1 and PCo2) explaining 68.3% and 14.7% of total variation, respectively (Figure 4a). Notably, all amended treatments were clearly distinguished from the CK, while BMFs containing different functional bacterial consortia also exhibited spatial separation within the ordination plot. The distinct clustering of CM away from the geometric center of BS, BM, AC, and RP in the PCoA ordination (Figure 4a) suggests that the four-strain consortium assembled into a community structure not predictable from the single-strain treatments alone. This likely reflects synergistic interactions among the inoculated strains—such as resource sharing, niche differentiation, and altered competition—that together reshaped the soil microbial community.

Complementary analysis through Venn diagrams elucidated microbial distribution patterns across treatments (Figure 4b). After filtering, a total of 372 unique ASVs were identified across all treatments in the Venn diagram analysis (Figure 4b), while the total ASV richness per sample ranged from 1,806 to 3,429, with a mean value of approximately 2,700 (Supplementary Table S1). Among 372 identified ASVs, 165 core ASVs (44.3%) persisted across all treatments. BMFs amended soils demonstrated significantly higher numbers of unique ASVs compared to CK.

Taxonomic distribution analysis at the phylum level revealed distinct treatment-induced microbial community restructuring patterns, as illustrated through distribution histograms (Figure 4c). The predominant bacterial phyla identified across all treatments comprised Firmicutes, Proteobacteria, Actinomycetota, Bacteroidetes, Gemmatimonadetes, Patescibacteria, Chloroflexi, Acidobacteriota, Myxococcota, and Planctomycetota. The amendments of BMFs induced marked phylum-level shifts: BS, BM, and CM preferentially enriched Firmicutes by 29.8%, 32.0%, and 34.1% respectively, while AC and RP amendments elevated Proteobacteria abundance by 27.8% and 23.5%. Gemmatimonadetes and Chloroflexi were slightly increased by the application of BS, BM, AC, and RP. In contrast, the relative abundances of Actinobacteriota and Myxococcota decreased upon the BMF amendments.

Class-level taxonomic analysis provided enhanced resolution of these microbial community dynamics (Figure 4d). Bacilli, the predominant class within the phylum Firmicutes, exhibited significant enrichment following BS, BM, and CM treatments. Gammaproteobacteria and Alphaproteobacteria, belonging to the phylum Proteobacteria, were significantly enhanced by the amendment of AC and RP, respectively. To further investigate how BMFs impact microbial communities, variations in key bacterial genera were analyzed. The relative abundance patterns of the 30 most prevalent genera are visualized in a hierarchically clustered heatmap (Figure 4e). The genus *Bacillus* (phylum: Firmicutes; class: Bacilli) showed higher relative abundances in BS, BM, and CM treatments compared to the CK group, consistent with the inoculation of *Bacillus subtilis* and *Bacillus megaterium*. Similarly, *Azotobacter* (phylum: Proteobacteria; class: Gammaproteobacteria) was more abundant in AC and CM groups, while *Rhodopseudomonas* (phylum: Proteobacteria; class: Alphaproteobacteria) dominated RP and CM soils.

These spatial distributions correspond directly to the functional taxa that have been introduced through biochar loading: *Bacillus subtilis, Bacillus megaterium, Azotobacter chroococcum, Rhodopseudomonas palustris*, and combined bacteria.

#### 3.4.3 Indicator microbes for each treatment

**Figure 5:**
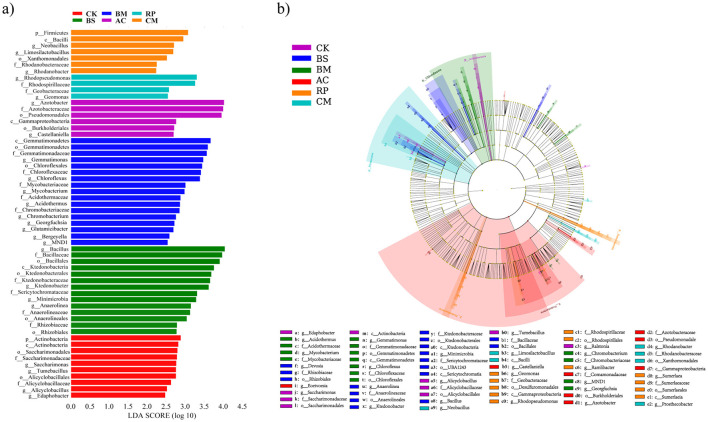
Taxonomic cladogram from LEfSe analysis showing differential abundance of soil microorganisms across different biochar-based microbial fertilizer treatments. **(a)** LDA score distribution of differentially abundant taxa (LDA > 2.0). **(b)** Cladogram showing the phylogenetic distribution of these taxa; circles from inside to outside represent phylum to genus, and colors indicate the treatment with significant enrichment.

A linear discriminant effect size analysis (LEfSe) was performed to identify and select distinctive bacterial taxa significantly associated with various BMFs, from the phylum to the genus level (Figures 5a, b). Unamended control harbored the genera *Saccharimonas* and *Alicyclobacillus*. The BS group contained four indicator genera: *Bacillus, Ktedonobacter, Anaerolinea*, and *Devosia*. The BM treatment was enriched with *Gemmatimonas, Chloroflexus, Mycobacterium, Acidothermus*, and *Chromobacterium*. The AC treatment was characterized by the genus *Azotobacter*. The RP treatment was characterized by the genera *Rhodopseudomonas* and *Sumerlaea*. The CM treatment was characterized by the genera *Neobacillus, Limosilactobacillus*, and *Rhodanobacter*.

#### 3.4.4 Functional prediction of microbial communities

**Figure 6:**
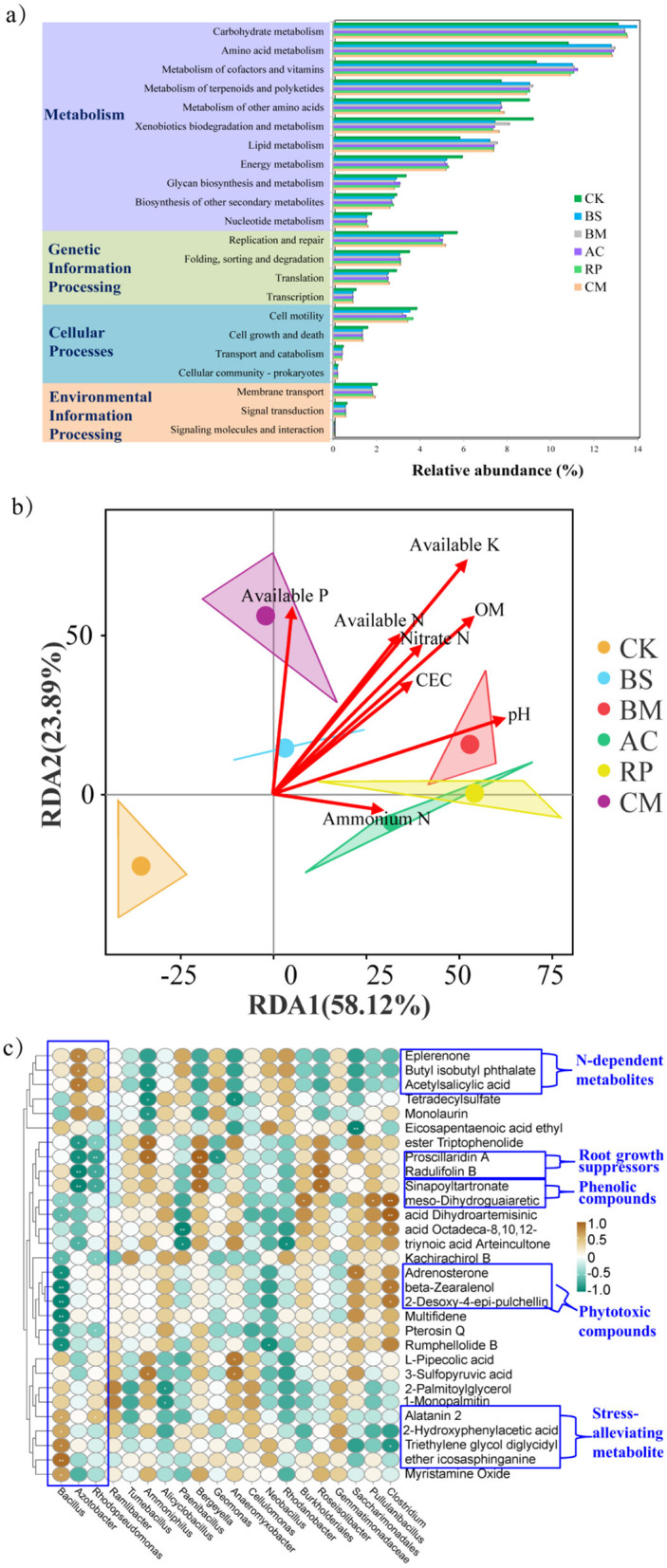
**(a)** Functional predictability of bacterial community; **(b)** Redundancy analysis (RDA) of bacterial community and selected environmental variables; **(c)** Correlation heatmap illustrating the relationship between metabolites and bacterial species. The color bar indicates Spearman's correlation coefficients ranging from −1 to 1. Significance levels: *p < 0.05 and **p < 0.01.

To elucidate the critical ecological functions of soil microbiota in response to BMFs, potential functions were predicted from amplicon data using the PICRUSt tool and Kyoto Encyclopedia of Genes and Genomes (KEGG) orthologs. Following the functional classification framework established by Yang and Zhang (2023), microbial functions were categorized into six major groups including Metabolism, Genetic Information Processing, Cellular Processes, Human Diseases, Environmental Information Processing, and Organismal Systems. The four predominant functional pathways, with their relative abundances ranked in descending order as metabolism, genetic information processing, cellular processes, and environmental information processing (Figure 6a). The application of BMFs significantly (*p* < 0.05) enhanced several metabolic pathways compared to CK, including carbohydrate and amino acid metabolism, cofactor and vitamin biosynthesis, terpenoid and polyketide metabolism, and lipid metabolism.

#### 3.4.5 Relationship between microbial community structure and soil properties

Redundancy analysis (RDA) was conducted on soil properties and microbial community structure to explore the key soil properties that drive the variations among microbial communities (Figure 6b). The statistical results describing the significance of each environmental factor are provided in Supplementary Table S2. Treatments exhibited strong spatial segregation along RDA1 (58.12%) and RDA2 (23.89%). BS, BM, AC, RP, and CM formed clusters which separated from the unamended CK. Furthermore, BMFs-amended treatments exhibited greater proximity to optimal soil property parameters compared to the unamended control, as demonstrated through multivariate clustering analysis. Notably, the microbial communities of BS and CM showed significant and positive correlations with available P, while pH, OM and ${\text{NH}}_{4}^{+}$-N were the major factors affecting grouped AC, BM and RP (Figure 6b).

### 3.5 BMFs regulated the rice rhizosphere soil metabolites

#### 3.5.1 Soil metabolites

Comprehensive untargeted metabolomic analysis of all soil samples identified 3,250 distinct metabolites, with detailed taxonomic classification and relative abundance distributions of these compounds presented in Supplementary Table S3. Lipids and lipid-like molecules emerged as the predominant class, constituting 28.31% of the total metabolites. Subsequent categories included organoheterocyclic compounds (15.72%), phenylpropanoids and polyketides (14.71%), organic acids and derivatives (14.34%), benzenoids (10.89%), organic oxygen compounds (8.18%), alkaloids and derivatives (2.22%), organic nitrogen compounds (1.85%), nucleosides, nucleotides, and analogs (1.32%), lignans, neolignans and related compounds (1.20%), and others (1.26%).

#### 3.5.2 Differentially expressed metabolites

The differentially expressed metabolites (DEMs) between the treatments were identified using the following criteria: VIP > 1.0 in the PLS-DA model and *p* < 0.05 in the *t*-test (Xiao et al., 2024). DEMs among the CK, BS, BM, AC, RP, and CM treatments were identified through pairwise comparisons and visualized using an UpSet plot (Supplementary Figure S2a). Supplementary Figure S2b further illustrates the significant up- and down-regulation of specific metabolites across treatment groups. Integrating data from Supplementary Figure S2a and b, 20 differential metabolites were identified in the CK vs. BS comparison (up = 1, down = 19), 13 differential metabolites in CK vs. BM (up = 10, down = 3), 175 differential metabolites in CK vs. AC (up = 38, down = 137), 35 differential metabolites in CK vs. RP (up = 18, down = 17), and 33 differential metabolites in CK vs. CM (up = 7, down = 26).

**Figure 7:**
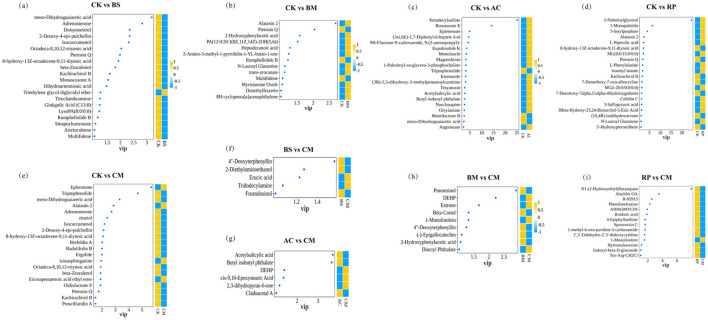
The bubble plots display the discriminating metabolites with a VIP value > 1.0 and significance level of *p* < 0.05; CK vs. BS **(a)**, CK vs. BM **(b)**, CK vs. AC **(c)**, CK vs. RP **(d)**, CK vs. CM **(e)**, BS vs. CM **(f)**, AC vs. CM **(g)**, BM vs. CM **(h)**, and RP vs. CM **(i)**. VIP indicates how much each metabolite contributed to the difference between the two groups.

The DEMs with ≥ two-fold upregulation or downregulation among pairwise comparison treatment groups are highlighted in Figure 7. The patterns and expression levels of DEMs were clearly visualized through the integration of VIP bubble plots and abundance heatmaps. In the CK vs. BS comparison, only triethylene glycol diglycidyl ether was upregulated, with all other metabolites showing downregulation (Figure 7a). As detailed in Supplementary Table S4, the downregulated metabolites were primarily categorized into lignans/neolignans, lipids/lipid-like molecules, benzenoids, and phenylpropanoids/polyketides, while the upregulated metabolite belonged to the organoheterocyclic compounds. Conversely, the CK vs. BM comparison revealed that three DEMs (pterosin Q, rumphellolide B, and multifidene) were downregulated, with the remainder upregulated (Figure 7b). The upregulated metabolites were classified as phenylpropanoids/polyketides, organic acids/derivatives, organoheterocyclic compounds, and organic nitrogen compounds, whereas downregulated metabolites were predominantly classified as benzenoids, lipids/lipid-like molecules, and hydrocarbons (Supplementary Table S5). In addition, the CK vs. AC comparison showed most DEMs were upregulated, with only triptophenolide, momilactone B, and meso-dihydroguaiaretic acid downregulated (Figure 7c). Upregulated classes comprised organic acids/derivatives, phenylpropanoids/polyketides, lipids/lipid-like molecules, benzenoids, and organic nitrogen compounds, while downregulated metabolites were lipids/lipid-like molecules, organoheterocyclic compounds, and lignans/neolignans (Supplementary Table S6). The CK vs. RP comparison exhibited 65% upregulated and 35% downregulated metabolites (Figure 7d). Upregulated categories mainly included lipids/lipid-like molecules, organoheterocyclic compounds, phenylpropanoids/polyketides, and organic acids/derivatives, while downregulated metabolites focused on lipids/lipid-like molecules, benzenoids, and phenylpropanoids/polyketides (Supplementary Table S7). In CK vs. CM comparison, only four DEMs (eplerenone, alatanin 2, icosasphinganine, eicosapentaenoic acid ethyl ester) were upregulated, with the rest downregulated (Figure 7e). Upregulated metabolites belonged to lipids/lipid-like molecules, phenylpropanoids/polyketides, and organic nitrogen compounds, while downregulated classes primarily included organoheterocyclic compounds, benzenoids, phenylpropanoids/polyketides, lipids/lipid-like molecules, and lignans/neolignans (Supplementary Table S8).

The DEMs exhibited significant overlap in pairwise comparisons. For instance, pterosin Q showed nearly universal significance in four major comparisons (CK vs. BM, CK vs. BS, CK vs. RP, CK vs. CM); as a benzenoid-class indane, it was consistently regulated and significantly reduced in the BS, BM, RP and CM treatments (Figure 7). Meso-dihydroguaiaretic acid also played a crucial role across three comparisons: CK vs. BS, CK vs. AC, and CK vs. CM. Kachirachirol B was similarly pivotal across three comparisons: CK vs. BS, CK vs. RP, and CK vs. CM. Adrenosterone, 2-desoxy-4-epi-pulchellin, and beta-zearalenol each played a prominent role in two comparisons: CK vs. BS and CK vs. CM. Alatanin 2 influenced differences in CK vs. BM, CK vs. RP, and CK vs. CM, while rumphellolide B and multifidene contributed to variations in CK vs. BS and CK vs. BM. Eplerenone and triptophenolide specifically impacted CK vs. AC and CK vs. CM comparisons.

#### 3.5.3 Enrichment analysis of metabolic pathways

**Figure 8:**
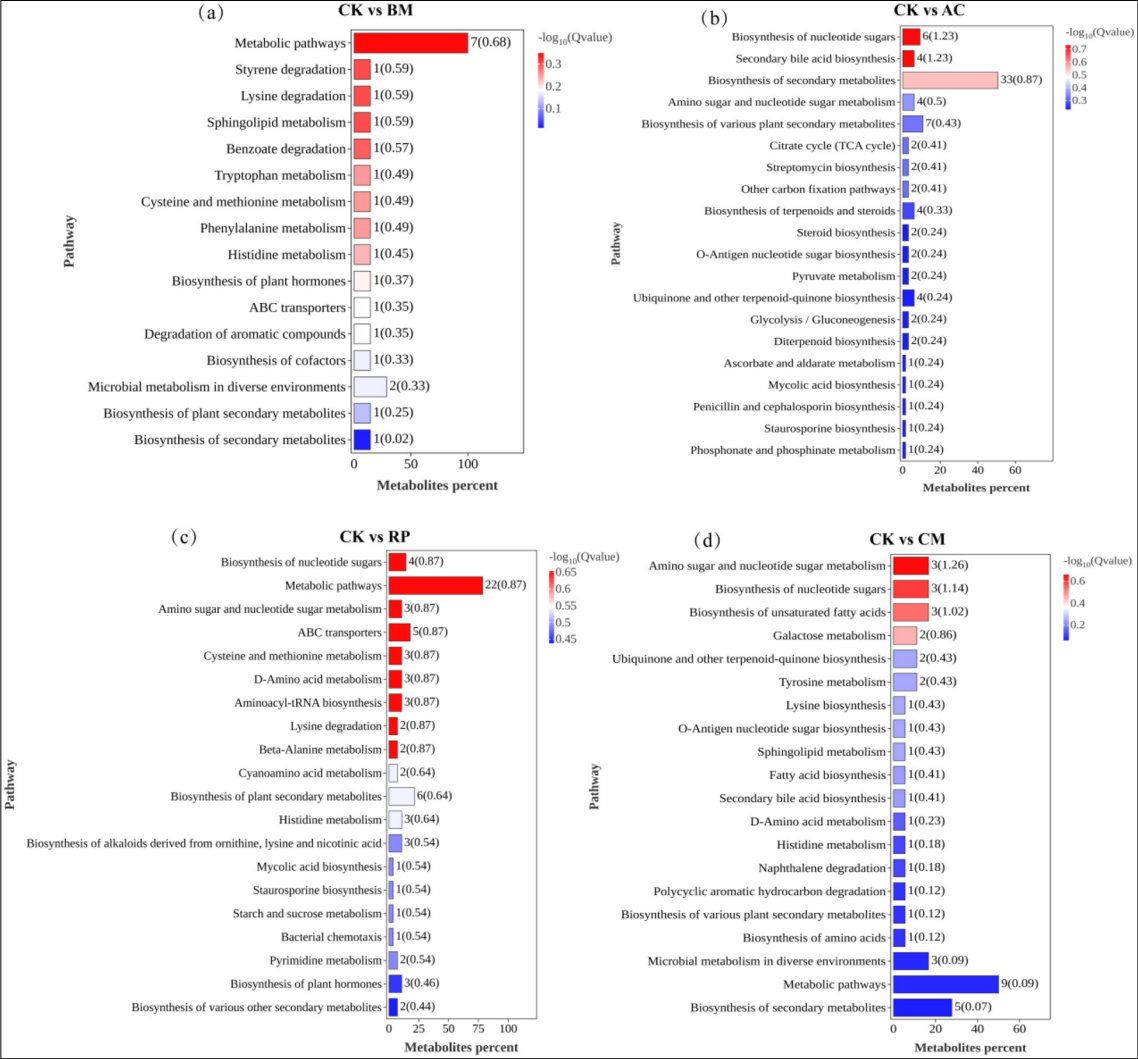
The top discriminating metabolic pathways in the CK vs. BM **(a)**, CK vs. AC **(b)**, CK vs. RP **(c)**, and CK vs. CM **(d)** comparisons.

Following the identification of DEMs, we analyzed their enriched metabolic pathways. KEGG pathway analysis unveiled key metabolic shifts within the differential metabolome, highlighting distinct treatment-specific patterns in six major categories: amino acid metabolism, carbohydrate metabolism, xenobiotics biodegradation and metabolism, lipid metabolism, terpenoid/polyketide metabolism, and cofactor/vitamin metabolism (Supplementary Figure S3). Compared to the CK group, the BM amendment notably altered metabolic pathways involved in styrene degradation, lysine degradation, benzoate degradation, and sphingolipid metabolism; the AC amendment significantly altered pathways for nucleotide sugar biosynthesis, secondary bile acid biosynthesis, secondary metabolite biosynthesis, and amino sugar and nucleotide sugar metabolism; the RP amendment markedly altered pathways governing nucleotide sugar biosynthesis, amino sugar and nucleotide sugar metabolism, cysteine and methionine metabolism, and D-amino acid metabolism; and the CM amendment affected amino sugar and nucleotide sugar metabolism, nucleotide sugar biosynthesis, and unsaturated fatty acid biosynthesis pathways (Figure 8).

### 3.6 Relationship between microbial communities and metabolites

Microbial communities play a crucial role in shaping soil metabolite distributions (Wang X. Y. et al., 2025; Xiao et al., 2024). To explore these interactions, Spearman's correlation analysis was conducted to assess associations between dominant bacterial genera and key differentially expressed metabolites (DEMs). The analysis revealed clear positive and negative correlations between specific microbial taxa and metabolite profiles (Figure 6c). Notably, the differentially expressed metabolites (DEMs) shown in Figure 6c were classified into four functional categories: stress-alleviating metabolites, phytotoxic compound suppressors, N-dependent metabolites, and root growth suppressors.

*Bacillus* exhibited significant positive correlations with icosasphinganine, alatanin 2, and triethylene glycol diglycidyl ether. Conversely, *Bacillus* showed significant negative correlations with mycotoxins (beta-zearalenol, adrenosterone) and phenolic/terpenoid metabolites (meso-dihydroguaiaretic acid, 2-desoxy-4-epi-pulchellin, rumphellolide B, kachirachirol B, and pterosin Q). *Azotobacter*, a N-fixing genus, was positively associated with eplerenone (a steroid), butyl isobutyl phthalate (a phthalate ester), and acetylsalicylic acid. Conversely, *Azotobacter* negatively correlated with terpenoids (triptophenolide, proscillaridin A) and phenolic metabolites (radulifolin B, sinapoyltartronate, and meso-dihydroguaiaretic acid). Similarly, *Rhodopseudomonas*, a phototrophic bacterium, displayed a significant positive correlation with alatanin 2. Conversely, this bacterium showed significant negative correlations with triptophenolide, proscillaridin A, radulifolin B, sinapoyltartronate, kachirachirol B, and pterosin Q.

## 4 Discussion

### 4.1 Mechanisms underlying the improvement of soil properties by BMFs

The improvement in soil quality under BMFs amendments is likely driven by the synergistic interplay of its primary components. The observed increase in soil pH can be attributed to the abundance of alkaline functional groups and basic cations present on the surface of biochar (Hou et al., 2024). The biochar and organic manure matrix collectively ameliorates the soil environment, wherein alkaline cations (e.g., Ca^2+^, Mg^2+^, K^+^) from biochar help neutralize acidity, while the OM directly enriches soil organic carbon pools and enhances CEC, thereby improving nutrient retention (Yang W. et al., 2021; Yang and Zhang, 2023). Concurrently, the inoculated functional microorganisms are inferred to mediate key biogeochemical processes, such as OM mineralization, biological N fixation, and P/K solubilization, collectively enhancing nutrient bioavailability (Bello et al., 2023; Wang et al., 2023). These processes are highly interconnected, as the improved soil conditions further support the activity and efficacy of the microbial consortium.

At the bacterial strain level, the functional roles within the consortium can be interpreted based on the known properties of the inoculated strains and their observed enrichment. For example, *Bacillus subtilis* secreted extracellular enzymes (sucrases, amylases, cellulases) that accelerated OM decomposition (Deng et al., 2025; Xia et al., 2025). *Bacillus megaterium* produced hydrolases such as alkaline phosphatase and phytase, facilitating the solubilization of recalcitrant P and K minerals (Qi et al., 2023). Additionally, *Azotobacter chroococcum* fixed atmospheric N_2_ into ${\text{NH}}_{4}^{+}$ via nitrogenase, thereby increasing ${\text{NH}}_{4}^{+}$-N content (Elakkya et al., 2025). Subsequently, the generated NH4? undergoes a series of oxidation reactions facilitated by nitrifying bacteria, resulting in the formation of nitrate (${\text{NO}}_{3}^{-}$) and an increase in ${\text{NO}}_{3}^{-}$-N levels (Li et al., 2024). *Rhodopseudomonas palustris*, as a representative photosynthetic bacterium, can produce phytohormones including auxins, gibberellins, and cytokinins, which can stimulate root growth, enhance nutrient uptake capacity, improve photosynthetic efficiency, and ultimately promote plant growth (Wang D. W. et al., 2022). *Rhodopseudomonas palustris* can also solubilize soil insoluble soil P and K by converting them into available forms (Xuan et al., 2024). Taking together, the combined treatment exhibited the greatest overall effect, with available N, P, and K increasing by 54.43%, 85.24%, and 50.40%, respectively, highlighting synergistic interactions among the four bacterial strains through complementary activities among the strains, rather than merely additive effects.

### 4.2 BMFs-induced changes in soil microbial communities and functions

Our results demonstrate that BMFs induce comprehensive changes in soil microbial properties, encompassing biomass, activity, community structure, and metabolic potential, which are intrinsically linked to the improved soil environment. The significant increases in microbial biomass C and N are attributed to the ability of BMFs to supply organic substrates and enhance nutrient availability, thereby promoting microbial growth (De Mastro et al., 2022). The stimulation of key enzyme activities (dehydrogenase, urease, sucrase, amylase, alkaline phosphatase) underscores the enhanced biochemical processing capacity of the soil. These enzymes catalyze critical steps in OM decomposition, N mineralization, and P solubilization, thereby accelerating nutrient cycling and improving soil fertility (Wang et al., 2023; Liu et al., 2022; El Fels et al., 2024). This is consistent with previous findings on biochar-based fertilizers (De Mastro et al., 2022; Palansooriya et al., 2025).

BMFs amendments also significantly increased microbial alpha-diversity and reshaped community structure. The proliferation of unique ASVs and distinct clustering in PCoA indicate that biochar-microbial complexes create niche-specific conditions that favor microbial colonization and diversification (Deng et al., 2025; Xing et al., 2024). Notably, treatments selectively enriched key functional phyla: BS, BM, and CM increased *Firmicutes* (notably class *Bacilli* and genus *Bacillus*), while AC specifically elevated *Proteobacteria* (primarily the class *Gammaproteobacteria* and the genus *Azotobacter*), and RP enriched *Proteobacteria* (mainly the class *Alphaproteobacteria* and the genus *Rhodopseudomonas*). *Firmicutes* and *Proteobacteria* are recognized as core functional groups in soil, driving C and N cycling and plant-microbe interactions (Zhao et al., 2021; Li et al., 2024; Wang et al., 2023). Their enrichment suggests BMFs enhance the functional capacity of the soil microbiome.

Remarkably, the inoculated strains (*Bacillus, Azotobacter, Rhodopseudomonas*) maintained ecological dominance after 120 days, which confirms the role of the biochar matrix in enhancing microbial persistence. Biochar's porous structure provides physical refuge and facilitates biofilm formation; its high surface area enables nutrient retention and slow release; and its improvement of soil conditions (pH, moisture, aggregation) creates a favorable niche that supports sustained colonization and activity of the inoculants (Qi et al., 2023; Wang et al., 2023; Feng et al., 2024; Deng et al., 2025).

Functional prediction revealed that Metabolism was the dominant category, with BMFs significantly enhancing pathways for carbohydrate, amino acid, and lipid metabolism. This indicates an overall upregulation of the microbial community's metabolic potential for processing C and N substrates, aligning with the observed improvements in nutrient cycling (Yang and Zhang, 2023; Hou et al., 2021; Gao et al., 2021). Redundancy analysis (RDA) further linked these microbial shifts to key soil properties (pH, OM, ${\text{NH}}_{4}^{+}$-N, available P), demonstrating that BMFs drive microbial community assembly by ameliorating the soil environment (Yan et al., 2021). The increased abundance of *Firmicutes* and *Proteobacteria*, which are crucial for soil biogeochemistry (Bello et al., 2023; Zhao et al., 2021), suggests a positive feedback loop where BMFs improve soil conditions, which in turn select for and stimulate microbial communities that further enhance soil fertility.

### 4.3 Microbial linkages to soil metabolite profiles

Soil metabolites were derived from microbial metabolites, plant root exudates, and the decomposition of plant residues (Dong et al., 2024). To elucidate how the core microbial genera (*Bacillus, Azotobacter*, and *Rhodopseudomonas*) coordinately regulate the metabolite pools classified as stress-alleviating metabolites, phytotoxic compound suppressors, N-dependent metabolites, and root growth suppressors, we discuss their specific correlations and potential underlying mechanisms.

The positive correlation of *Bacillus* with icosasphinganine, alatanin 2, and triethylene glycol diglycidyl ether likely reflects its metabolic capabilities. Icosasphinganine synthesis likely occurs through *Bacillus* pathways involving serine palmitoyltransferase, important for membrane integrity and signaling (Stein, 2005). Alatanin 2, a terpenoid, probably originates from *Bacillus*-specific non-ribosomal peptide synthetase or polyketide synthase systems (Saalim et al., 2024). The positive correlation with triethylene glycol diglycidyl ether suggests *Bacillus* utilizes this synthetic epoxy compound as a carbon source, degrading it via hydrolytic enzymes such as epoxide hydrolases into glycols for energy metabolism (Win et al., 2020). Conversely, its negative correlations with mycotoxins and phenolic/terpenoid metabolites suggest active degradation or detoxification through enzymes like esterases and laccases, either detoxifying toxins or utilizing them as energy sources (Liu et al., 2023).

For *Azotobacter*, the positive associations with eplerenone, butyl isobutyl phthalate, and acetylsalicylic acid highlight its role in N-dependent metabolic pathways, as exemplified by eplerenone-a steroid that requires N for heterocyclic ring synthesis (Wang J. et al., 2025). *Azotobacter* degrades phthalate esters such as butyl isobutyl phthalate via meta-cleavage pathways using phthalate dioxygenases and hydrolases, converting them into tricarboxylic acid (TCA) cycle intermediates as carbon sources (Wang X. et al., 2022). Additionally, this genus hydrolyzes acetylsalicylic acid to salicylic acid, potentially using it as a carbon source or signaling molecule (Xu et al., 2023). Conversely, its negative correlations with terpenoids and phenolic metabolites may stem from two factors: the oxidation of terpenoids by superoxide radicals generated during nitrogen fixation (N fixation), which disrupts their structural integrity, and competition with plants and fungi for phenylalanine—a key precursor in phenolic biosynthesis—thus limiting the synthesis of phenolic compounds (Papaianni et al., 2025).

Similarly, for *Rhodopseudomonas*, the positive link to alatanin 2 aligns with its phototrophic capacity to fuel terpenoid biosynthesis via photosynthetic generation of ATP and NADPH (Klaus et al., 2022). Its broad negative correlations with phenolic/terpenoid metabolites are likely mediated through light-induced reactive oxygen species (ROS), which oxidize phenolic groups and cleave unsaturated bonds in these compounds (Panigrahy et al., 2022). It also secretes laccases and peroxidases to degrade phenolics like sinapoyltartronate and pterosin Q (Rabiee et al., 2024). Furthermore, its photo-driven metabolism creates anoxic microzones, inhibiting aerobic fungi responsible for terpenoid synthesis (Klaus et al., 2022).

Collectively, these findings highlight the distinct metabolic capacities and ecological functions of *Bacillus, Azotobacter*, and *Rhodopseudomonas*, as reflected in their specific metabolite associations. The combined action of these taxa within microbial consortia may further amplify their effects through functional complementarity, leading to synergistic enhancement of metabolic pathways. Overall, the results demonstrate a strong interconnection between soil microbial communities and metabolomic profiles, underscoring the pivotal role of soil microbes in driving diverse biochemical processes essential for ecosystem functioning and crop productivity.

### 4.4 Integrated mechanism of BMFs on soil fertility and rice productivity

**Figure 9:**
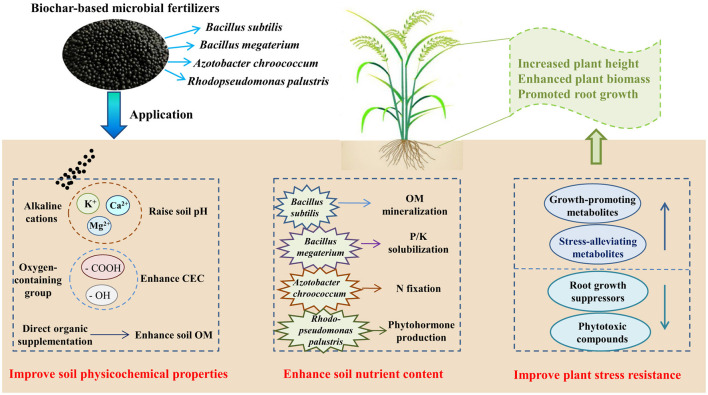
Synergistic mechanisms of biochar-based microbial fertilizers in enhancing soil fertility and rice productivity.

The application of BMFs markedly enhanced soil fertility by improving pH, OM, CEC, and the availability of N, P, and K, thereby promoting rice growth. These improvements can be attributed to synergistic interactions between biochar and functional microbial traits, as revealed by integrated metabolomic and biogeochemical analyses (Figure 9).

First, alkaline cations (Ca^2+^, Mg^2+^, K^+^) in biochar exchange with soil-bound H^+^ and Al^3+^, neutralizing acidity to raise pH (Yang W. et al., 2021). This pH adjustment creates ideal conditions for *Bacillus* and *Azotobacter*, whose enzymatic activities (e.g., laccases, nitrogenases) are highly pH-sensitive (Wang et al., 2023). Second, the inoculated microbial consortium collectively enhanced nutrient bioavailability by driving key processes: *Bacillus* strains (including *Bacillus subtilis* and *Bacillus megaterium*) contribute to OM decomposition via sucrases, amylases, and cellulases (Fu et al., 2023) and enhance nutrient availability through the solubilization of recalcitrant P and K minerals by secreting alkaline phosphatase and phytase (Zhao et al., 2021); *Azotobacter chroococcum* fixes atmospheric N_2_ into ${\text{NH}}_{4}^{+}$ via nitrogenase, with subsequent nitrification to ${\text{NO}}_{3}^{-}$ (Li et al., 2024); and *Rhodopseudomonas palustris* solubilizes insoluble P and K (Khuong et al., 2020) while producing phytohormones (auxins, cytokinins, gibberellins) to stimulate photosynthesis (Wang D. W. et al., 2022). Third, the rich organic matter in these fertilizers directly supplements soil nutrients, enhances soil OM and CEC, improves nutrient retention, and reduces leaching losses—ensuring steady availability of ${\text{NH}}_{4}^{+}$-N, ${\text{NO}}_{3}^{-}$-N, and mineral ions (Yang and Zhang, 2023). Finally, biochar enhances soil structural stability by promoting macroaggregate formation through microbial exopolysaccharide production, thereby facilitating root penetration and optimizing water uptake efficiency (Hou et al., 2021).

Notably, microbial-metabolite crosstalk functionally underpins these processes: *Bacillus* enrichment positively correlates with stress-alleviating metabolites (icosasphinganine, alatanin 2) while suppressing phytotoxic compounds (adrenosterone, beta-zearalenol, 2-Desoxy-4-epi-pulchellin); *Azotobacter* associates with N-dependent metabolites (eplerenone, butyl isobutyl phthalate, and acetylsalicylic acid) and reduces phenolics (sinapoyltartronate, meso-dihydroguaiaretic acid); *Rhodopseudomonas* links to alatanin 2 while inhibiting root growth suppressors (proscillaridin A, radulifolin B) (Figure 9). This metabolite-mediated regulation enhances microbial functionality and plant stress resilience. Stress alleviation, combined with improved nutrient access, translates into significantly greater rice growth compared to the control.

Taken together, biochar integrated with complementary microbial consortia creates a synergistic system that improves soil chemistry, nutrient cycling (N fixation, P/K solubilization, OM mineralization), and metabolite regulation, thereby enhancing rice growth and resilience. The sustained activity of inoculated microbes ensures long-term fertility improvements while suppressing phytotoxins. These insights provide a framework for designing next-generation biofertilizers to support sustainable crop production in degraded soils. However, while our results clearly demonstrate the overall benefits of the integrated BMF system, we cannot definitively attribute the observed effects to the inoculated microbes vs. the carrier matrix. Future studies with a complete set of controls (biochar alone, manure alone, functional microbial fertilizer alone, and carrier without microbes) are needed to separate out the effects of each component and their synergistic interactions.

## 5 Conclusions

This study demonstrates that biochar-based microbial fertilizers (BMFs) restore soil fertility and enhance rice productivity through driving a comprehensive improvement in soil health—including alleviating acidification, improving CEC, and increasing OM and nutrient (N, P, K) availability—which led to promoted rice growth. 16S rRNA gene sequencing and metabolomic analyses suggest that these benefits are associated with synergistic interactions within the BMF system, including biochar-mediated soil conditioning, microbial-driven nutrient cycling, and metabolite-assisted plant–soil feedback regulation. Notably, the co-enrichment of functional genera such as *Bacillus, Azotobacter*, and *Rhodopseudomonas* was closely linked to improvements in nutrient availability and the production of plant-growth-promoting metabolites, supporting a collective role in OM mineralization, nutrient solubilization, and phytohormone synthesis. The microbial-metabolite networks not only supported nutrient acquisition but also suppressed phytotoxic compounds, thereby improving root development and plant resilience. These findings highlight BMFs as a transformative alternative to chemical fertilizers, offering a sustainable pathway to address soil degradation while reducing reliance on synthetic inputs. Additionally, our results provide a scientific foundation for developing targeted biochar and microbe formulations for different crop systems. Future efforts integrating genomics, long-term impact assessment, and field-scale validation will be crucial to accelerate the translation of BMFs into global sustainable agriculture.

## Data Availability

The raw sequencing data (16S rRNA) is available in the NCBI Sequence Read Archive (SRA) under BioProject accession number PRJNA1435571, and the metabolomic data is available in the MetaboLights repository under accession number MTBLS14036.
