# Supplementary material for: Biochar-based microbial fertilizer improves soil fertility and rice productivity by regulating soil nutrient-microbe-metabolite interactions
Source: Front Microbiol. 2026 Mar 30;17:1802769. doi: 10.3389/fmicb.2026.1802769 (PMC13071027; doi:10.3389/fmicb.2026.1802769)
Supplement: Supplementary file 1 [file Supplementary_file_1.docx]

**Supplementary Material**

**Biochar-based microbial fertilizer improves soil fertility and rice productivity by regulating soil nutrient-microbe-metabolite interactions**

Wendan Xiao^a^; Dan Li^b^; Qi Zhang^a^; De Chen^a^; Zhen Zhao^a^; Miaojie Huang^a^; Xiaolei Huang^a^; Xuezhu Ye^a,*^

**Institutions:**

^a^ State Key Laboratory for Quality and Safety of Agro-Products; Key Laboratory of Soil Remediation and Quality Improvement of Zhejiang Province, Institute of Environmental Resources, Soil and Fertilizer, Zhejiang Academy of Agricultural Sciences, Hangzhou 310021, China

^b^ Hangzhou Agricultural Technology Extension Center (Hangzhou Plant Protection and Quarantine Center), Hangzhou 310019, China

***Corresponding author:**

Dr. Xuezhu Ye

Tel: +86-0571-86415206; Fax: +86-0571-86419052

E-mail: yexz@zaas.ac.cn**Section 1. Characterization of soil**

Soil pH was measured using a glass electrode pH meter. OM was quantified by potassium dichromate oxidation and titration (Morona et al., 2017). CEC was determined using the neutral ammonium acetate method (Mattila and Rajala, 2022). Total N was measured using the micro-Kjeldahl method (Calazans et al., 2018). Available N was quantified using the alkali solution diffusion method (Yan et al., 2021). Available P was extracted with 0.5 M NaHCO_3_ solution, and available K with 1 M NH_4_AC solution. After filtration, P and K contents were measured using an automatic chemical analyzer (Smartchem 200, Italy) and an atomic absorption spectrometer (ContrAA 700, Germany). NH_4_^+^-N and NO_3_^−^-N were extracted with 1 M KCl (1:5 solid/liquid ratio) and quantified using a segmented flow analyzer (Skalar San++, France) (Sraj et al., 2014).

**References:**

Calazans, S.O.L., Morais, V.A., Scolforo, J.R.S., Zinn, Y.L., Mello, J.M., Mancini, L.T., Silva, C.A., 2018. Soil organic carbon as a key predictor of N in forest soils of Brazil. Journal of Soils and Sediments 18, 1242-1251.

Mattila, T.J., Rajala, J., 2022. Estimating cation exchange capacity from agronomic soil tests: Comparing Mehlich-3 and ammonium acetate sum of cations. Soil Science Society of America Journal 86, 47-50.

Morona, F., Melquíades, F.L., Muller, M.M.L., 2017. Quantification of Organic Matter in Agricultural Soils from the Central Region of Parana State, Brazil. Communications in Soil Science and Plant Analysis 48, 2288-2293.

Sraj, L.O., Almeida, M., Swearer, S.E., Kolev, S.D., McKelvie, I.D., 2014. Analytical challenges and advantages of using flow-based methodologies for ammonia determination In estuarine and marine waters. Trac-Trends in Analytical Chemistry 59, 83-92.

Yan, T.T., Xue, J.H., Zhou, Z.D., Wu, Y.B., 2021. Biochar-based fertilizer amendments improve the soil microbial community structure in a karst mountainous area. Science of the Total Environment, 794, 148757.

**Section 2. 16S rRNA gene amplification and sequencing**

**1）PCR amplification**

The *16S rRNA* gene V3-V4 region was amplified by PCR (95 °C for 5 min; 30 cycles of 95 °C for 1 min, 60 °C for 1 min, 72 °C for 1 min; final extension at 72 °C for 7 min) using primers 341F (5'-CCTACGGGNGGCWGCAG-3') and 806 R (5'-GGACTACHVGGGTATCTAAT -3'). The 50 μL PCR mixture included 10 μL 5 × Q5® Reaction Buffer, 10 μL 5 × Q5® High GC Enhancer, 1.5 μL 2.5 mM dNTPs, 1.5 μL 10 μM primers, 0.2 μL Q5@ High-Fidelity DNA Polymerase, and 50 ng template DNA.

**2）Illumina NovaSeq 6000 sequencing**

Amplicons were extracted from 2% agarose gels, purified using an AxyPrep DNA Gel Extraction Kit (Axygen Biosciences, Union City, CA, U.S.), and quantified using ABI StepOnePlus Real-Time PCR System (Life Technologies, Foster City, USA). Purified amplicons were pooled in equimolar amounts and paired-end sequenced (PE250) on an Illumina platform. Raw reads were deposited in the NCBI Sequence Read Archive (SRA) database.

**3）Bioinformatics analysis**

The DADA2 package in QIIME2 software removed low-quality reads (quality score < 25) and chimeric sequences. DADA2 generated amplicon sequence variants (ASVs) with ≥100 % similarity. ASVs were taxonomically classified using the SILVA database and a naive Bayesian model based on the RDP classifier (version 2.2). Relative abundance of ASVs was normalized at each taxonomic level.

Taxon abundance was visualized using Krona (version 2.6). Community composition was visualized as a stacked bar plot using the R project ggplot2 package (version 2.2.1). Species abundance was plotted in circular layouts using Circos (version 0.69-3). A heatmap of species abundance was plotted using the pheatmap package (version 1.0.12) in R. Pearson’s correlation analysis was conducted using R project psych package (version 1.8.4). Venn analysis was performed using R project VennDiagram package (version 1.6.16). Shannon, Simpson, Chao1, ACE, and Good’s coverage indices were calculated using QIIME (version 1.9.1). Principal coordinate analysis (PCoA) was generated using R project Vegan package (version 2.5.3) and plotted using ggplot2 package. Bacterial ecological functions were profiled using the FAPROTAX database (v1.0).

**Section 3. Soil metabolite analysis using UHPLC-QE-MS**

Soil samples (1 g) were extracted in 5 mL Eppendorf tube with 1 mL of 1:1 acetonitrile: methanol containing 10 μL of adonitol (0.5 mg mL^-1^) as an internal standard, vortexed for 30 s, sonicated in ice water for 10 min, and incubated at -40 °C for 1 h to precipitate proteins. After centrifugation at 12000 rpm at 4 °C for 15 min, the supernatant was analyzed by ultra-high performance liquid chromatography Q-Exactive^TM^ HF mass spectrometry (UHPLC-QE-MS) in positive or negative ion mode. In positive ion mode, Eluent A was 0.1% formic acid in water, and eluent B was methanol. In negative ion mode, eluent A was 5 mM ammonium acetate (pH 9.0), and eluent B was methanol. The solvent gradient was: 2% B (0 to 1.5 min), 2%−100% B (1.5 min to 12 min), 100% B (12 to 14 min), 100%–2% B (14 to 14.1 min), and 2% B (14.1 to 17 min). The Q-Exactive^TM^ HF mass spectrometer operated with a spray voltage of 3.2 kV, capillary temperature of 320 °C, sheath gas flow rate of 40 arb, and auxiliary gas flow rate of 10 arb.

**Table S1** Summary of sequence tags and ASVs.

| Sample ID | Total Tags | Taxon Tags | Unclassified Tags | Singleton Tags | ASVs |
| --- | --- | --- | --- | --- | --- |
| CK-1 | 104307 | 104307 | 0 | 0 | 3214 |
| CK-2 | 83028 | 83026 | 2 | 0 | 2664 |
| CK-3 | 105645 | 105645 | 0 | 0 | 3032 |
| BS-1 | 104625 | 104625 | 0 | 0 | 2775 |
| BS-2 | 100687 | 100685 | 2 | 0 | 3018 |
| BS-3 | 99172 | 99172 | 0 | 0 | 2910 |
| BM-1 | 106982 | 106982 | 0 | 0 | 2723 |
| BM-2 | 105127 | 105127 | 0 | 0 | 2885 |
| BM-3 | 111238 | 111238 | 0 | 0 | 3429 |
| AC-1 | 102661 | 102661 | 0 | 0 | 2415 |
| AC-2 | 96454 | 96454 | 0 | 0 | 2065 |
| AC-3 | 104629 | 104629 | 0 | 0 | 2671 |
| RP-1 | 94858 | 94858 | 0 | 0 | 2455 |
| RP -2 | 98384 | 98384 | 0 | 0 | 1806 |
| RP -3 | 109065 | 109061 | 4 | 0 | 2480 |
| CM-1 | 100497 | 100497 | 0 | 0 | 2393 |
| CM-2 | 107079 | 107079 | 0 | 0 | 3010 |
| CM-3 | 100300 | 100298 | 2 | 0 | 3270 |
| Average | 101929 | 101929 | 0 | 0 | 2734 |

**Table S2** Statistical summary of RDA results.

| **Environmental variable** | **R²** | **p-value** | **Significance** |
| --- | --- | --- | --- |
| pH | 0.5841 | 0.012 | * |
| OM | 0.6794 | 0.008 | ** |
| CEC | 0.3628 | 0.065 | ns |
| Ammonium N | 0.1625 | 0.156 | ns |
| Nitrate N | 0.4345 | 0.033 | * |
| Available N | 0.4377 | 0.031 | * |
| Available P | 0.4568 | 0.025 | * |
| Available K | 0.5243 | 0.018 | * |

**Table S3** Classification of differential metabolites in all the treatment groups and their total percentage.

| **Superclass metabolites** | **Total number** | **Percentage (%)** |
| --- | --- | --- |
| Lipids and lipid-like molecules | 920 | 28.31 |
| Organoheterocyclic compounds | 511 | 15.72 |
| Phenylpropanoids and polyketides | 478 | 14.71 |
| Organic acids and derivatives | 466 | 14.34 |
| Benzenoids | 354 | 10.89 |
| Organic oxygen compounds | 266 | 8.18 |
| Alkaloids and derivatives | 72 | 2.22 |
| Organic nitrogen compounds | 60 | 1.85 |
| Nucleosides, nucleotides, and analogues | 43 | 1.32 |
| Lignans, neolignans and related compounds | 39 | 1.20 |
| Organohalogen compounds | 12 | 0.37 |
| Organosulfur compounds | 9 | 0.28 |
| Hydrocarbons | 8 | 0.25 |
| Hydrocarbon derivatives | 6 | 0.18 |
| Homogeneous non-metal compounds | 1 | 0.03 |
| Acetylides | 1 | 0.03 |
| Mixed metal/non-metal compounds | 1 | 0.03 |
| Organic 1,3-dipolar compounds | 1 | 0.03 |
| Organometallic compounds | 1 | 0.03 |
| Organophosphorus compounds | 1 | 0.03 |

**Table S4** Summary of the DEMs profiles and their up- and down-regulation in the CK vs. BS treatment groups.

| **Metabolite** | **VIP** | **P value** | **Regulate** | **M/Z** | **Retention time** | **Class** |
| --- | --- | --- | --- | --- | --- | --- |
| Meso-Dihydroguaiaretic acid | 3.19 | 0.045708 | down | 353.17 | 6.445 | Lignans, neolignans and related compounds |
| Adrenosterone | 2.83 | 0.049223 | down | 301.18 | 6.191 | Lipids and lipid-like molecules |
| Doisynoestrol | 2.35 | 0.040476 | down | 299.16 | 6.078 | Lipids and lipid-like molecules |
| 2-Desoxy-4-epi-pulchellin | 2.35 | 0.030332 | down | 233.15 | 6.063 | Lipids and lipid-like molecules |
| Isocurcumenol | 2.32 | 0.027048 | down | 235.17 | 6.322 | Lipids and lipid-like molecules |
| Octadeca-8,10,12-triynoic acid | 2.03 | 0.028548 | down | 273.19 | 6.233 | Lipids and lipid-like molecules |
| Pterosin Q | 1.98 | 0.008488 | down | 251.13 | 5.582 | Benzenoids |
| 8-hydroxy-13Z-octadecene-9,11-diynoic acid | 1.92 | 0.035072 | down | 291.20 | 6.095 | Lipids and lipid-like molecules |
| Beta-Zearalenol | 1.80 | 0.037902 | down | 303.16 | 6.072 | Phenylpropanoids and polyketides |
| Kachirachirol B | 1.60 | 0.028601 | down | 313.14 | 5.777 | Phenylpropanoids and polyketides |
| Monascusone A | 1.53 | 0.043705 | down | 253.11 | 5.624 | Organoheterocyclic compounds |
| Dihydroartemisinic acid | 1.47 | 0.048889 | down | 237.19 | 6.664 | Lipids and lipid-like molecules |
| Triethylene glycol diglycidyl ether | 1.24 | 0.012558 | up | 261.13 | 5.699 | Organoheterocyclic compounds |
| Tiruchanduramine | 1.22 | 0.015831 | down | 323.16 | 6.573 | Organoheterocyclic compounds |
| Ginkgolic Acid (C13:0) | 1.22 | 0.016314 | down | 343.22 | 6.506 | Benzenoids |
| LysoPA(8:0/0:0) | 1.20 | 0.032673 | down | 299.13 | 5.734 | Lipids and lipid-like molecules |
| Rumphellolide B | 1.14 | 0.00366 | down | 253.18 | 6.299 | Lipids and lipid-like molecules |
| Sinapoyltartronate | 1.05 | 0.049097 | down | 325.06 | 5.052 | Phenylpropanoids and polyketides |
| Arteincultone | 1.01 | 0.046353 | down | 267.16 | 6.041 | Organoheterocyclic compounds |
| Multifidene | 1.01 | 0.003727 | down | 149.13 | 7.262 | Hydrocarbons |

**Table S5** Summary of the DEMs profiles and their up- and down-regulation in the CK vs. BM treatment groups.

| **Metabolite** | **VIP** | **P value** | **Regulate** | **M/Z** | **Retention time** | **Class** |
| --- | --- | --- | --- | --- | --- | --- |
| Alatanin 2 | 2.70 | 0.0123 | up | 832.24 | 11.42 | Phenylpropanoids and polyketides |
| Pterosin Q | 2.05 | 0.0196 | down | 251.13 | 5.58 | Benzenoids |
| 2-Hydroxyphenylacetic acid | 1.58 | 0.0488 | up | 153.05 | 0.58 | Benzenoids |
| PA(12:0/20:3(8Z,11Z,14Z)-2OH(5,6)) | 1.52 | 0.0148 | up | 675.42 | 9.76 | - |
| Heptadecanoic acid | 1.28 | 0.0269 | up | 269.25 | 10.90 | Lipids and lipid-like molecules |
| 2-Amino-3-methyl-1-pyrrolidin-1-YL-butan-1-one | 1.25 | 0.0170 | up | 171.15 | 5.96 | Organic acids and derivatives |
| Rumphellolide B | 1.22 | 0.0063 | down | 253.18 | 6.30 | Lipids and lipid-like molecules |
| N-Lauroyl Glutamine | 1.12 | 0.0354 | up | 329.24 | 11.50 | Organic acids and derivatives |
| trans-urocanate | 1.12 | 0.0074 | up | 139.05 | 1.46 | Organoheterocyclic compounds |
| Multifidene | 1.05 | 0.0163 | down | 149.13 | 7.26 | Hydrocarbons |
| Myristamine Oxide | 1.04 | 0.0499 | up | 258.28 | 7.49 | Organic nitrogen compounds |
| Dimethylfraxetin | 1.01 | 0.0413 | up | 259.06 | 5.82 | Phenylpropanoids and polyketides |
| 8H-cyclopenta[a]acenaphthylene | 1.00 | 0.0136 | up | 191.09 | 7.57 | Benzenoids |

**Table S6** Summary of the DEMs profiles and their up- and down-regulation in the CK vs. AC treatment groups.

| **Metabolite** | **VIP** | **P value** | **Regulate** | **M/Z** | **Retention time** | **Class** |
| --- | --- | --- | --- | --- | --- | --- |
| Tetradecylsulfate | 25.14 | 0.0002 | up | 293.18 | 8.68 | Organic acids and derivatives |
| Broussonin E | 14.23 | 0.0031 | up | 311.13 | 7.61 | Phenylpropanoids and polyketides |
| Eplerenone | 4.91 | 0.0142 | up | 437.19 | 6.76 | Lipids and lipid-like molecules |
| (3xi,6E)-1,7-Diphenyl-6-hepten-3-ol | 4.51 | 0.0189 | up | 265.16 | 7.44 | Phenylpropanoids and polyketides |
| 9H-Fluorene-9-carboxamide, 9-(3-aminopropyl) | 4.29 | 0.0085 | up | 265.13 | 7.52 | Benzenoids |
| Eupalinolide K | 3.66 | 0.0075 | up | 361.17 | 8.82 | Lipids and lipid-like molecules |
| Monolaurin | 3.44 | 0.0009 | up | 297.20 | 8.13 | Lipids and lipid-like molecules |
| Magnoshinin | 3.36 | 0.0134 | up | 415.21 | 6.76 | Lignans, neolignans and related compounds |
| 1-Palmitoyl-sn-glycero-3-phosphocholine | 3.31 | 0.0435 | up | 496.34 | 9.62 | Lipids and lipid-like molecules |
| Triptophenolide | 3.31 | 0.0342 | down | 313.18 | 6.44 | Lipids and lipid-like molecules |
| Irtemazole | 3.22 | 0.0275 | up | 289.14 | 7.37 | Organoheterocyclic compounds |
| (3R)-3,5-dihydroxy-3-methylpentanoylcarnitine | 3.22 | 0.0227 | up | 292.18 | 5.55 | Lipids and lipid-like molecules |
| Tinyatoxin | 2.89 | 0.0074 | up | 599.26 | 7.79 | Lipids and lipid-like molecules |
| Acetylsalicylic acid | 2.84 | 0.0265 | up | 163.04 | 7.62 | Benzenoids |
| Butyl isobutyl phthalate | 2.81 | 0.0239 | up | 279.16 | 7.61 | Benzenoids |
| Norclozapine | 2.79 | 0.0039 | up | 313.12 | 7.58 | Organoheterocyclic compounds |
| Octylamine | 2.67 | 0.0036 | up | 130.16 | 5.33 | Organic nitrogen compounds |
| Momilactone B | 2.64 | 0.0094 | down | 329.18 | 6.44 | Organoheterocyclic compounds |
| meso-Dihydroguaiaretic acid | 2.49 | 0.0104 | down | 353.17 | 6.45 | Lignans, neolignans and related compounds |
| Argenteane | 2.32 | 0.0027 | up | 655.33 | 8.68 | Lignans, neolignans and related compounds |

**Table S7** Summary of the DEMs profiles and their up- and down-regulation in the CK vs. RP treatment groups.

| **Metabolite** | **VIP** | **P value** | **Regulate** | **M/Z** | **Retention time** | **Class** |
| --- | --- | --- | --- | --- | --- | --- |
| 2-Palmitoylglycerol | 24.47 | 0.0478 | up | 353.27 | 10.01 | Lipids and lipid-like molecules |
| 1-Monopalmitin | 6.39 | 0.0373 | up | 331.28 | 10.02 | Lipids and lipid-like molecules |
| 5-borylporphine | 4.03 | 0.0192 | up | 321.13 | 5.81 | Organoheterocyclic compounds |
| Alatanin 2 | 2.58 | 0.0304 | up | 832.24 | 11.42 | Phenylpropanoids and polyketides |
| L-Pipecolic acid | 2.42 | 0.0048 | up | 152.07 | 5.01 | Organic acids and derivatives |
| 8-hydroxy-13Z-octadecene-9,11-diynoic acid | 2.26 | 0.0241 | down | 291.20 | 6.10 | Lipids and lipid-like molecules |
| MG(0:0/15:0/0:0) | 2.17 | 0.0419 | up | 339.25 | 9.56 | Lipids and lipid-like molecules |
| Pterosin Q | 1.93 | 0.0199 | down | 251.13 | 5.58 | Benzenoids |
| L-Phenylalanine | 1.90 | 0.0036 | up | 166.09 | 5.05 | Organic acids and derivatives |
| Isoamyl laurate | 1.86 | 0.0366 | up | 271.26 | 10.02 | Lipids and lipid-like molecules |
| Kachirachirol B | 1.68 | 0.0486 | down | 313.14 | 5.78 | Phenylpropanoids and polyketides |
| 7-Demethoxy-7-oxo-albocycline | 1.61 | 0.0436 | down | 293.17 | 6.06 | Phenylpropanoids and polyketides |
| MG(i-20:0/0:0/0:0) | 1.46 | 0.0277 | up | 387.35 | 7.37 | Lipids and lipid-like molecules |
| 7-Deacetoxy-7alpha,11alpha-dihydroxygedunin | 1.44 | 0.0375 | down | 457.22 | 6.26 | Lipids and lipid-like molecules |
| Cohibin C | 1.40 | 0.0212 | up | 577.52 | 10.00 | Lipids and lipid-like molecules |
| 3-Sulfopyruvic acid | 1.38 | 0.0230 | up | 168.98 | 4.76 | Organic acids and derivatives |
| 3Beta-Hydroxy-23,24-Bisnorchol-5-Enic Acid | 1.30 | 0.0418 | up | 369.24 | 10.01 | Lipids and lipid-like molecules |
| (1S,4R)-isodihydrocarvone | 1.20 | 0.0395 | down | 353.05 | 5.22 | - |
| N-Lauroyl Glutamine | 1.16 | 0.0231 | up | 329.24 | 11.50 | Organic acids and derivatives |
| 3'-Hydroxypterostilbene | 1.16 | 0.0366 | down | 273.11 | 5.58 | Phenylpropanoids and polyketides |

**Table S8** Summary of the DEMs profiles and their up- and down-regulation in the CK vs. CM treatment groups.

| **Metabolite** | **VIP** | **P value** | **Regulate** | **M/Z** | **Retention time** | **Class** |
| --- | --- | --- | --- | --- | --- | --- |
| Eplerenone | 5.67 | 0.032 | up | 437.19 | 6.76 | Lipids and lipid-like molecules |
| Triptophenolide | 4.68 | 0.033 | down | 313.18 | 6.44 | Lipids and lipid-like molecules |
| meso-Dihydroguaiaretic acid | 3.32 | 0.018 | down | 353.17 | 6.45 | Lignans, neolignans and related compounds |
| Alatanin 2 | 2.87 | 0.024 | up | 832.24 | 11.42 | Phenylpropanoids and polyketides |
| Adrenosterone | 2.72 | 0.048 | down | 301.18 | 6.19 | Lipids and lipid-like molecules |
| zinniol | 2.40 | 0.041 | down | 289.14 | 5.85 | Benzenoids |
| Isocurcumenol | 2.19 | 0.028 | down | 235.17 | 6.32 | Lipids and lipid-like molecules |
| 2-Desoxy-4-epi-pulchellin | 2.13 | 0.039 | down | 233.15 | 6.06 | Lipids and lipid-like molecules |
| 8-hydroxy-13Z-octadecene-9,11-diynoic acid | 2.09 | 0.021 | down | 291.20 | 6.10 | Lipids and lipid-like molecules |
| Brefeldin A | 2.03 | 0.050 | down | 263.16 | 6.23 | Phenylpropanoids and polyketides |
| Radulifolin B | 2.01 | 0.040 | down | 277.14 | 5.87 | Lipids and lipid-like molecules |
| Ergolide | 2.01 | 0.043 | down | 307.15 | 5.82 | Organoheterocyclic compounds |
| icosasphinganine | 1.95 | 0.046 | up | 330.34 | 8.12 | Organic nitrogen compounds |
| Octadeca-8,10,12-triynoic acid | 1.92 | 0.035 | down | 273.19 | 6.23 | Lipids and lipid-like molecules |
| beta-Zearalenol | 1.90 | 0.027 | down | 303.16 | 6.07 | Phenylpropanoids and polyketides |
| Eicosapentaenoic acid ethyl ester | 1.90 | 0.044 | up | 353.25 | 10.02 | Lipids and lipid-like molecules |
| Oidiolactone F | 1.89 | 0.042 | down | 295.15 | 5.88 | Organoheterocyclic compounds |
| Pterosin Q | 1.76 | 0.013 | down | 251.13 | 5.58 | Benzenoids |
| Kachirachirol B | 1.52 | 0.033 | down | 313.14 | 5.78 | Phenylpropanoids and polyketides |
| Proscillaridin A | 1.52 | 0.050 | down | 513.28 | 6.49 | Lipids and lipid-like molecules |

**Fig. S1** The effective bacterial number of the biochar-based microbial fertilizers.

Note: BS: *Bacillus subtilis* loaded biochar-based microbial fertilizer; 3) BM: *Bacillus megaterium* loaded biochar-based microbial fertilizer; 4) AC: *Azotobacter chroococcum* loaded biochar-based microbial fertilizer; 5) RP: *Rhodopseudomonas palustris* loaded biochar-based microbial fertilizer; 6) CM: biochar-based microbial fertilizer loaded with combined bacteria.


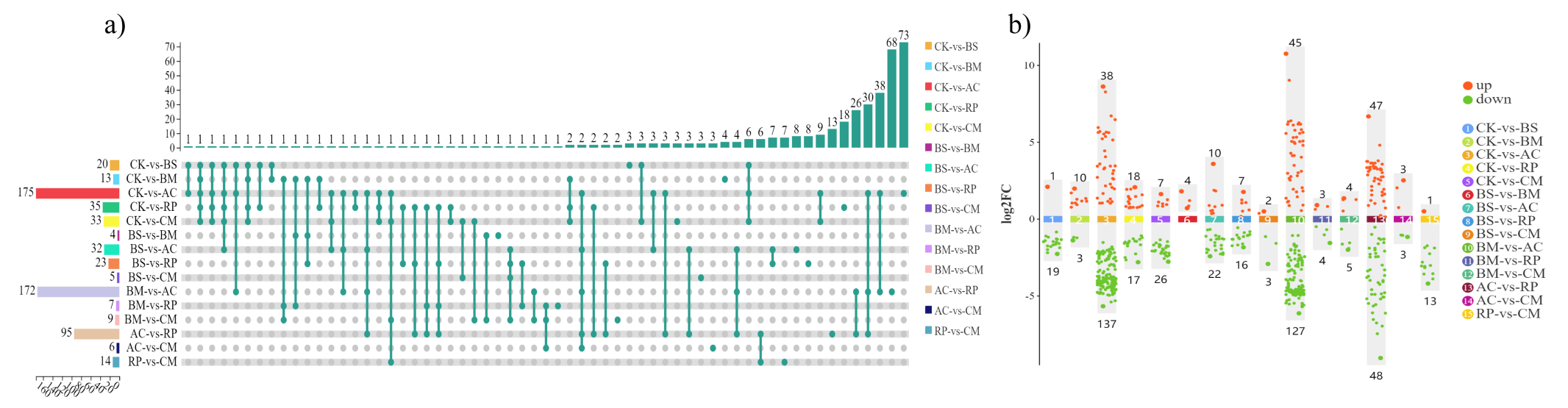


**Fig. S2** Upset plot showing the comparative analysis of soil differential metabolites between different groups.


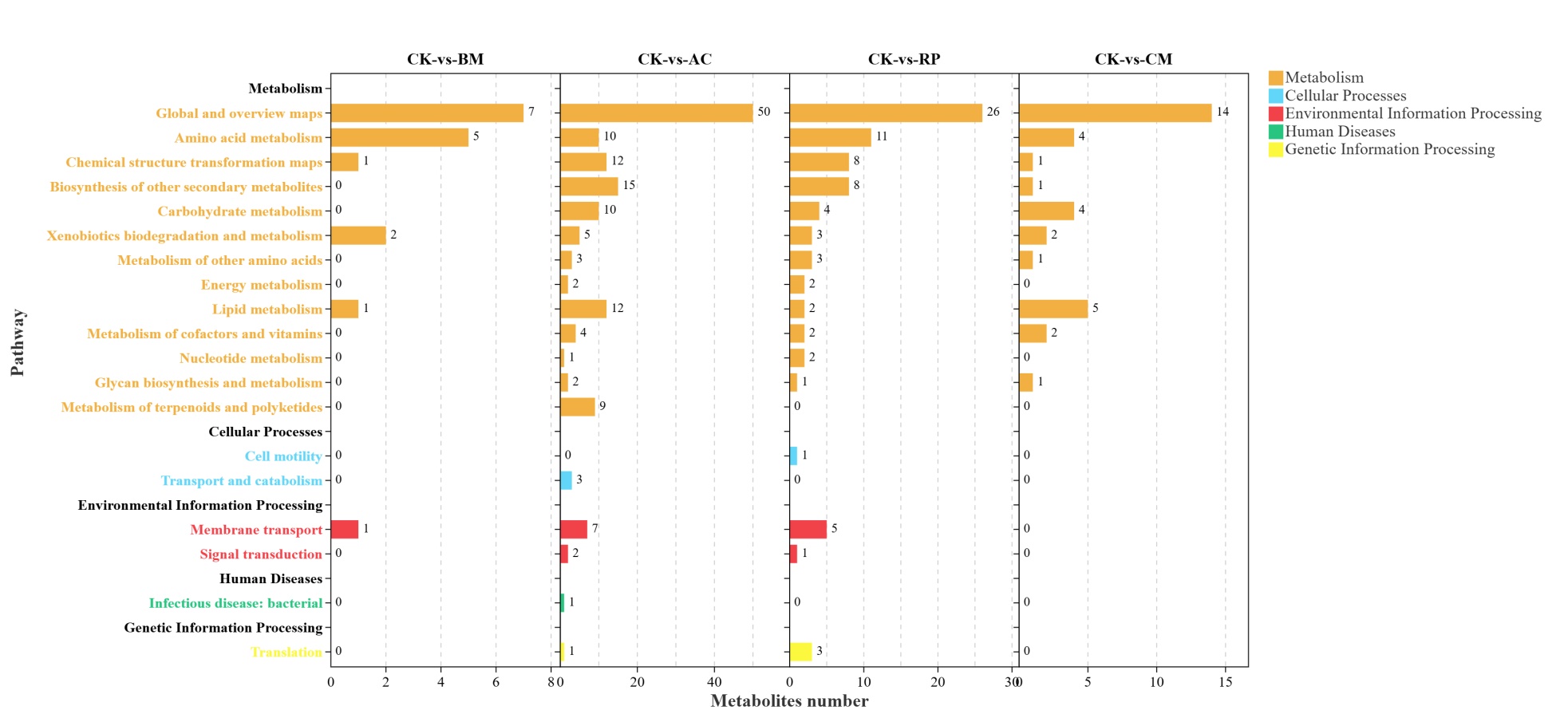


**Fig. S3** KEGG metabolic pathways in different treatment groups. The vertical coordinate is the classification of the KEGG metabolic pathway and the horizontal coordinate is the number of metabolites annotated to the pathway.
